# Pd Intercalation in BiOCl Nanosheets Promotes Ambient Electrosynthesis of Urea: Operando Study by Synchrotron X‑ray Spectroscopies

**DOI:** 10.1021/acsami.6c03918

**Published:** 2026-05-06

**Authors:** Yu-Chung Chang, Shao-Hua Yu, Ruei-Hung Juang, Hsing-Ye Chen, Aline Wong Taladua, Cheng-Shiuan Li, Rey Yonson Capangpangan, Chun-Hong Kuo

**Affiliations:** † Department of Applied Chemistry, 34914 National Yang Ming Chiao Tung University, Hsinchu 300093, Taiwan; ‡ Department of Chemistry, 69330 Mindanao State University - Iligan Institute of Technology, Iligan 9200, Philippines; § Green Energy and Environment Research Laboratories, 63129 Industrial Technology Research Institute, Hsinchu 310401, Taiwan; ∥ Department of Physical Sciences and Mathematics, College of Fisheries and Marine Sciences, Mindanao State University at Naawan, Naawan 9023, Philippines; ⊥ Center for Emergent Functional Matter Science, National Yang Ming Chiao Tung University, Hsinchu 300093, Taiwan; # National Synchrotron Radiation Research Center, Hsinchu 300092, Taiwan

**Keywords:** urea, BiOCl, intercalation, operando spectroscopy, X-ray absorption spectroscopy

## Abstract

Electrochemical urea synthesis offers an energy-efficient route for converting CO_2_ and NO_3_^–^ into a high-demand nitrogen commodity. However, urea selectivity hinges on orchestrating the coformation and interfacial coupling of CO_2_-derived *CO_*x*_ and N-reduction *NH_*x*_ intermediates. Consequently, achieving high urea rates and Faradaic efficiencies requires optimized catalyst designs. Here, we engineered 2D BiOCl nanosheets with Pd^0^ to promote C–N coupling while suppressing H_2_ evolution and Bi^0^ segregation under negative potentials. We hydrothermally synthesized BiOCl and Pd-containing BiOCl nanosheets, both of which serve as electrocatalysts for the electrosynthesis of urea. Structural characterization confirmed that the Pd species are intercalated Pd^0^ sub-nm clusters within the interlayers of the BiOCl nanosheets. The maximum Faradaic efficiency for urea reached 12.43 ± 1.02% over Pd–BiOCl and 6.99 ± 1.62% over BiOCl at −0.30 V vs RHE. Operando experiments were conducted using synchrotron powder X-ray diffraction and X-ray absorption spectroscopy, enabling real-time observation of phase transitions and local coordination environments. These results revealed that CO_3_^2–^ intercalation occurred in both BiOCl and Pd-BiOCl nanosheets, leading to the formation of Bi_2_O_2_CO_3_. However, the layered framework of the nanosheets cracked at −0.5 V or at more negative potentials as Bi^0^ segregated and aggregated into nanoclusters. In contrast, BiOCl nanosheets with intercalated Pd^0^ subnm clusters avoided this issue over the range −0.1– −0.5 V. From these results, we conclude that intercalating Pd subnm clusters into BiOCl nanosheets stabilizes the layered framework, suppresses Bi^0^ segregation, lowers the charge-transfer resistance, and enhances anion exchange toward urea production within the BiOCl interlayers.

## Graphical abstract

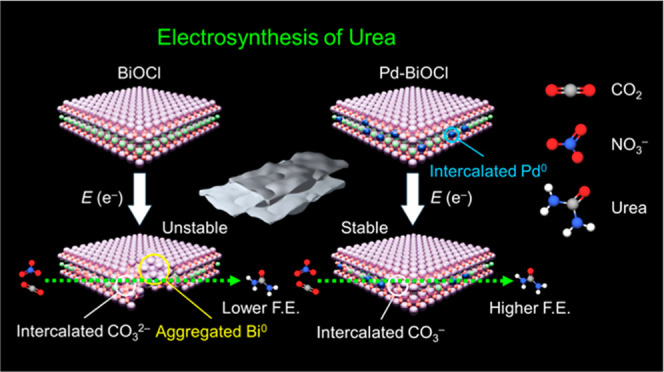

## Introduction

Urea is a cornerstone industrial feedstock and the most widely used nitrogen fertilizer.^1^ In modern industry, urea production relies on a two-step process: energy-intensive ammonia synthesis via the Haber–Bosch reaction (N_2_ + 3H_2_ → 2NH_3_), followed by thermochemical conversion of NH_3_ and CO_2_ through ammonium carbamate formation and subsequent dehydration to urea (2NH_3_ + CO_2_ → CO­(NH_2_)_2_ + H_2_O, the Bosch–Meiser process), typically operated at elevated temperatures (150–200 °C) and pressures (150–250 bar).^2–4^ While the urea-forming step itself is thermodynamically favorable, the overall process is intrinsically constrained by the high energy demand and carbon footprint of upstream ammonia synthesis. Collectively, ammonia-based fertilizer production is estimated to consume ∼2% of global energy and contribute ∼1.4% of anthropogenic CO_2_ emissions, underscoring the urgency of developing alternative, lower-carbon nitrogen utilization pathways.^5^ In this context, electrochemical urea synthesis has recently emerged as a promising strategy, enabling the direct coupling of CO_2_ with nitrogen-containing species under ambient conditions and potentially bypassing or complementing conventional ammonia production while overcoming the limitations of the traditional thermochemical pathway.^6–8^ However, urea formation is kinetically complex and highly selective, requiring the synchronized generation and interfacial coupling of CO_2_-derived *CO_*x*_ and nitrogen-reduction-derived *NH_*x*_ intermediates.^9–11^ Achieving efficient C–N bond formation and high Faradaic efficiency, therefore, demands catalysts capable of simultaneously regulating adsorption energetics, charge transfer, and the local reaction microenvironment, including pH, bicarbonate/carbonate speciation, and mass transport. Therefore, rationally designed heterostructures that spatially integrate CO_2_- and N-activation sites have attracted significant attention. In recent years, catalyst design for urea electrosynthesis has advanced through oxygen-vacancy engineering,^12–14^ doping,^15^ bimetallic catalysts,^16–18^ and single-atom catalysts (SACs),^19–21^ each targeting the formation and coupling of key intermediates. For example, Wei et al. showed that CeO_2_ nanorods with tunable oxygen vacancies served as an effective platform for stabilizing CO_2_-derived intermediates and facilitating charge transfer.^12^ Similarly, Lv et al. employed a doping engineering strategy to synthesize a SrCo_0.39_Ru_0.61_O_3−δ_ catalyst, demonstrating enhanced C–N coupling efficiencies.^15^ Collectively, these insights have motivated the rational design of heterostructures that spatially integrate CO_2_-activation and N-activation sites to promote interfacial CO_*x*_–NH_*x*_ coupling, thereby enhancing both reaction activity and product selectivity. Two-dimensional bismuth oxyhalides (BiOX; X = Cl, Br, I) are layered tetragonal materials consisting of [X–Bi–O–Bi–X] slabs separated by van der Waals gaps.^22^ Their exposed facets can host oxygen vacancies that modulate the local electronic structure and adsorption, thereby stabilizing CO_2_-derived intermediates and enhancing N_2_ adsorption/activation toward NH_3_.^23^ Meanwhile, Bi-based oxides have also been recognized as efficient catalysts for CO_2_RR due to their weak H* binding, high formate selectivity, low toxicity, and corrosion resistance, which collectively suppress the competing hydrogen evolution reaction (HER).^24,25^ Our previous work has shown that both BiOCl and Pd-BiOCl nanosheets acted as precatalysts and were readily converted into their active states under applied potentials. The active states of the nanosheets involved the formation of nanoscale Bi^0^ islands on their surfaces, which drove CO_2_ reduction to formate. In addition, intercalating Pd subnm clusters into the layers of BiOCl (Pd-BiOCl) reduced the charge-transfer barrier and enhanced the formate yield rate in CO_2_RR.^26^

In this work, we provide insights into the abilities and mechanisms of BiOCl and Pd-BiOCl nanosheets in the electrosynthesis of urea via the coreduction of CO_2_ and NO_3_^–^. Figure 1 presents a schematic summarizing the significant findings. In brief, we hydrothermally synthesized BiOCl and Pd-containing BiOCl nanosheets, both of which serve as electrocatalysts for urea electrosynthesis. Structural characterizations confirmed that the Pd species were intercalated Pd subnm clusters. For urea production, Pd-BiOCl nanosheets delivered a Faradaic efficiency (F.E.) of 12.43 ± 1.02% at −0.30 V vs RHE, outperforming pristine BiOCl (6.99 ± 1.62%) and commercial Pd/C (0%). Since only Pd/C generated CO gas and did not produce urea, the intercalated Pd^0^ subnm clusters were regarded as playing an auxiliary role rather than serving as the active sites for urea formation. Through operando experiments using synchrotron powder X-ray diffraction (SPXRD) and X-ray absorption spectroscopy (SXAS), we found that CO_3_^2–^ intercalation occurred in BiOCl nanosheets between −0.2 and −0.4 V, leading to the formation of Bi_2_O_2_CO_3_. However, the layered framework of the nanosheets cracked at −0.5 V or more negative potentials as Bi^0^ segregated and aggregated into clusters (Figure 1a). In contrast, BiOCl nanosheets with intercalated Pd^0^ subnm clusters maintained an intact BiOCl layered framework over −0.1 to −0.5 V and triggered CO_3_^2–^ intercalation beginning at −0.1 V (Figure 1b). We therefore infer that intercalating Pd^0^ subnm clusters into BiOCl nanosheets contributes to stabilizing the layered framework, mitigating the reduction to Bi^0^, lowering the charge-transfer resistance, and enhancing anion exchange toward C–N coupling within the BiOCl interlayers.^26–28^

**Figure 1:**
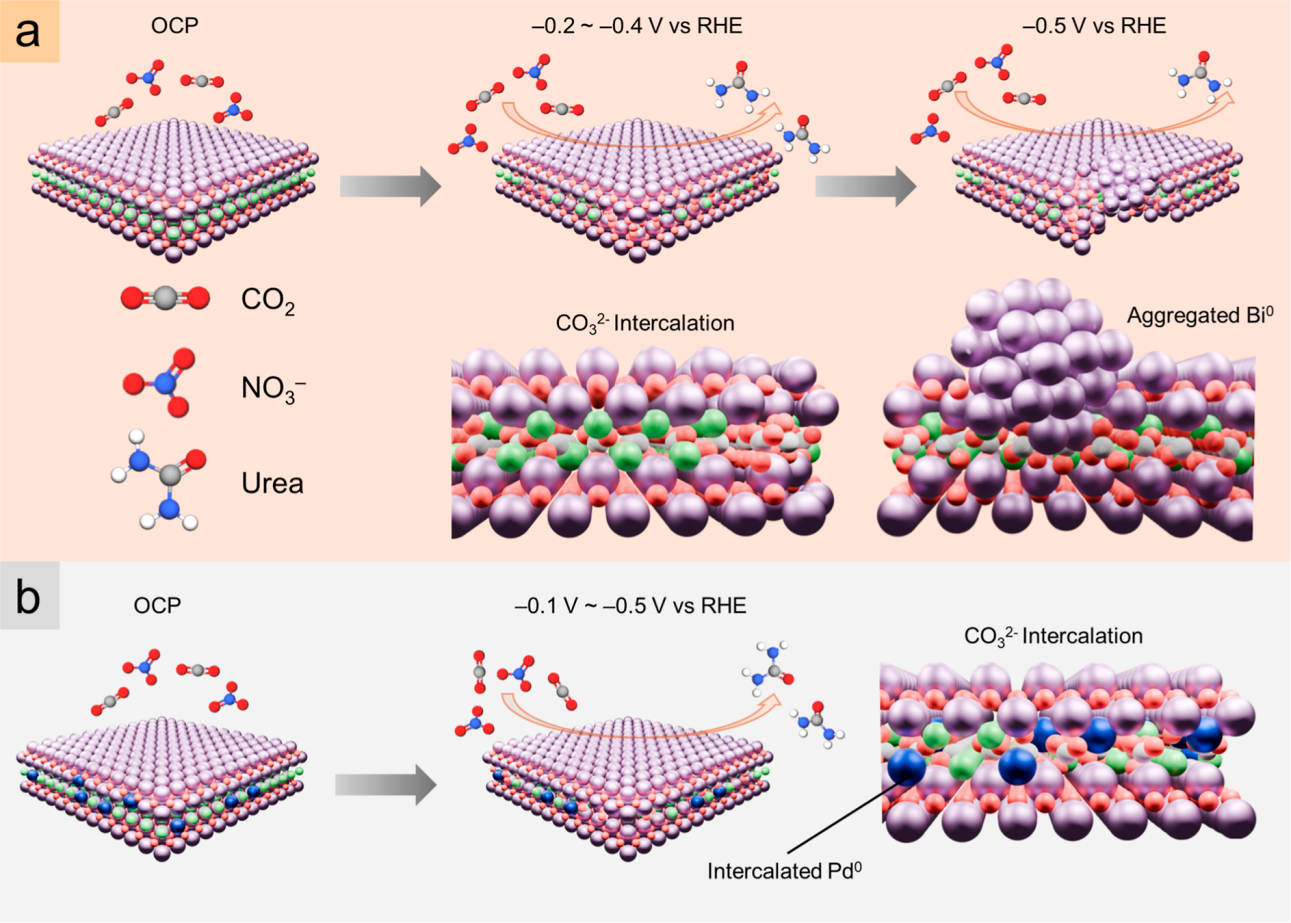
Schematic illustration of urea electrosynthesis catalyzed by (a) BiOCl and (b) Pd-intercalated BiOCl nanosheets.

## Experimental Section

### Chemicals

Potassium chloride (KCl, 99.0–100.5%, J. T. Baker), bismuth­(III) nitrate pentahydrate (Bi­(NO_3_)_3_·5H_2_O, 99.95%, Sigma-Aldrich), D-sorbitol (C_6_H_14_O_6_, 98%, Sigma-Aldrich), palladium­(II) sodium chloride (Na_2_PdCl_4_, 99.95%, Thermo Fisher Scientific), potassium bicarbonate (KHCO_3_, 100.1%, J. T. Baker), potassium nitrate (KNO_3_, 98%, Sigma-Aldrich), carbon dioxide (^12^CO_2_, 99.999%, Ching Fong Gas Industrial), potassium nitrite (KNO_2_, ≥ 96.0%, Sigma-Aldrich). Nafion solution (C_7_HF_13_O_5_S·C_2_F_4_, 5 wt % in lower aliphatic alcohols/water, 15–20% water content, Sigma-Aldrich), palladium on carbon (Pd/C, 10 wt %, Sigma-Aldrich), carbon paper (100 × 50 mm^2^, 0.18 mm thick, Clean Energy Technology), N-(1-naphthyl)­ethylenediamine (C_12_H_14_N_2_, > 98%, Sigma-Aldrich), sulfanilamide (H_2_NC_6_H_4_SO_2_NH_2_, ≥ 98%, Alfa Aesar), phosphoric acid (H_3_PO_4_, 85%, J. T. Baker), salicylic acid (C_7_H_6_O_3_, 99%, Sigma-Aldrich), sodium citrate dihydrate (C_6_H_5_Na_3_O_7_·5H_2_O, > 98%, Sigma-Aldrich), sodium hydroxide (NaOH, ≥ 99%, Sigma-Aldrich), sodium perchlorate (NaClO_4_, ≥ 98%, Sigma-Aldrich), sodium nitroprusside dihydrate (Na_2_[Fe­(CN)_5_NO]·2H_2_O, ≥ 99%, Sigma-Aldrich), urease (300 unit/mg, TCI), ammonium chloride (NH_4_Cl, 99.5%, Sigma-Aldrich). Deionized water (DI H_2_O, 18.2 MΩ·cm) was used in all solution preparations. All chemicals were used as received without further purification.

### Synthesis of BiOCl Nanosheets

Typically, Bi­(NO_3_)_3_·5H_2_O (162 mg, 0.334 mmol), D-sorbitol (30 mg, 0.165 mmol), and KCl (25 mg, 0.335 mmol) were dissolved in 10 mL of DI H_2_O under vigorous stirring for 15 min until the solution became clear. The initial pH was adjusted to 1.0–1.5 with dilute nitric acid (HNO_3_). The solution was then transferred to a 25 mL Teflon-lined stainless-steel autoclave and heated to 160 °C with a ramping rate of 3 °C/min. After the temperature reached 160 °C and stabilized, heating was maintained for 6 h, after which the autoclave was allowed to cool naturally to room temperature. The resulting white suspension was collected by centrifugation at 8,000 rpm for 5 min. The supernatant was removed, and the collected colloids were redispersed in 10 mL of a mixed ethanol/DI H_2_O solution, followed by centrifugation for washing. This washing step was repeated three times, and the final product was dried overnight in a vacuum oven at 50 °C.

### Synthesis of Pd-BiOCl Nanosheets

Pd-BiOCl nanosheets were synthesized using the same procedure as above, except that Na_2_PdCl_4_ was added to the precursor solution before hydrothermal heating. In brief, 162 mg (0.33 mmol) Bi­(NO_3_)_3_·5H_2_O, 30 mg (0.16 mmol) sorbitol, 16.2 mg Na_2_PdCl_4_ (ca. 10 wt %), and 25 mg (0.34 mmol) KCl were dissolved in 10 mL of DI water. The solution was then transferred to a thick-walled PTFE jar and heated at 160 °C for 6 h in an air-circulated oven. After cooling to room temperature, the resulting white suspension was collected by centrifugation at 8000 rpm for 5 min. The collected colloids were washed and centrifuged three additional times, and the final product was dried overnight under vacuum at 50 °C to remove the solvent.

### Characterization

For scanning electron microscopy (SEM) and transmission electron microscopy (TEM) characterization, 5 μL of the sample suspension was drop-cast onto a 0.3 × 0.3 cm^2^ silicon wafer (for SEM) or a copper grid (for TEM), and the samples were slowly dried at room temperature. SEM images were obtained using a Hitachi SU-8010 microscope operated at 15 kV. Bright-field TEM and high-angle annular dark-field scanning TEM (HAADF-STEM) images, along with energy-dispersive X-ray spectroscopy (EDS) mapping, were recorded on an FEI Talos F200XG microscope (Thermo Fisher Scientific) equipped with a Super-X G2 EDS detector and operated at 200 kV. Elemental compositions were determined by inductively coupled plasma mass spectrometry (ICP-MS, Thermo ELEMENT XR). Synchrotron powder X-ray diffraction patterns were collected at beamline TPS 19A of the National Synchrotron Radiation Research Center (NSRRC), using an incident X-ray energy of 20 keV (λ = 0.61992 Å). Powder samples were loaded into glass capillaries (outer diameter 0.30 mm, wall thickness 0.01 mm, initial length 80 mm; HR-610, Hampton Research). A packed powder bed of approximately 10 mm was formed at one end by gentle tapping to promote uniform compaction. Each capillary was then trimmed with a diamond scribe to a final total length of 20 mm, and the open end was sealed with clay. Atomic force microscopy (AFM) images and height profiles were recorded using a Hitachi AFM-100 with a silicon probe. High-resolution X-ray photoelectron spectroscopy (HR-XPS) was performed on a ULVAC-PHI PHI Quantera II spectrometer equipped with a scanning X-ray microprobe and an Al Kα source. Gaseous products were quantified using an Agilent 8890 gas chromatograph (GC) equipped with both a thermal conductivity detector (TCD, Ar carrier gas) and a flame ionization detector (FID). Liquid products were analyzed using a JASCO V-670 UV–Vis spectrophotometer. Nuclear magnetic resonance (NMR) spectra were recorded in water-gated mode on a JEOL JNM-ECZ500R/S1 spectrometer (500 MHz).

### Electrochemical Measurements

All measurements were conducted at room temperature using a CHI760E potentiostat in a two-compartment H-cell separated by a Nafion 117 proton exchange membrane (Figure S1). A Pt wire served as the counter electrode (CE), and a saturated calomel electrode (SCE, sat. KCl) was used as the reference electrode (RE). The working electrode (WE) was a piece of carbon paper (CP, 1.0 × 0.5 cm^2^) loaded with Pd/C nanoparticles, BiOCl, or Pd-BiOCl nanosheets as electrocatalysts. To load the nanosheets, a catalyst ink was prepared by ultrasonically dispersing 2.0 mg of nanosheets in 40 μL of Nafion solution (5 wt %) and 100 μL of isopropanol for 20 min. Then, 1.0 mg of the ink was drop-cast onto both sides of the CP (0.5 mg per side) to achieve a loading density of 2.0 mg cm^–2^. The resulting electrodes are denoted as BiOCl-WE, Pd-BiOCl-WE, and Pd/C-WE for comparison. The working electrode was mounted in a PTFE holder, with the CP in contact with a flat Pt pad. All applied potentials were converted to values versus the reversible hydrogen electrode (RHE).

1 $$E_{RHE}=E_{SCE}+0.241+0.0591\times pH$$

For the electrosynthesis of urea, both the anode and cathode compartments of the H-cell were filled with 18 mL of a 0.1 M KNO_3_/KHCO_3_ electrolyte saturated with CO_2_ (pH = 6.80 ± 0.03). A constant CO_2_ flow of 20 mL min^–1^ was supplied during the measurements. In one control experiment, only CO_2_ reduction was carried out in a CO_2_-saturated 0.1 M KHCO_3_ electrolyte by excluding 0.1 M KNO_3_. In another control experiment, an Ar-saturated electrolyte of 0.1 M KHCO_3_ + 0.1 M KNO_3_ (pH = 8.80 ± 0.09) was used for urea electrosynthesis.

The electrochemical double-layer capacitance (*C*_dl_) was determined by recording cyclic voltammograms (CV_s_) within a potential range corresponding to a nonfaradaic process at scan rates (r) of 20, 40, 60, 80, and 100 mV s^–1^. The potential windows for CV scans were defined as the open-circuit potential (OCP) ± 0.05 V vs RHE. The double-layer capacitance (*C*_dl_), equal to one-half of d­(Δ*I*)/d­(r), was then estimated by plotting ΔIOCP = (*I*_top_ – *I*_bottom_ at OCP) as a function of the scan rate (*r*). The electrochemically active surface areas (ECSAs) were calculated as follows.

2 $$ECSA=C_{dl}/C_{s}$$

*C*_s_ is the specific capacitance of a smooth surface in the same electrolyte. In the ECSA calculation, *C*_s_ denotes the specific capacitance of a soft, planar electrode with an actual surface area of 1 cm^2^, typically taken as 0.04 mF·cm^–2^. Electrochemical impedance spectroscopy (EIS) was performed at an applied potential of −0.3 V vs RHE, with an AC perturbation amplitude of 5 mV, over a frequency range of 100 kHz–0.1 Hz. Durability tests for urea electrosynthesis were performed at −0.3 V vs RHE under constant potential conditions for 100 h.

The Faradaic efficiency (F.E.) is defined as the ratio of the number of electrons consumed in forming a specific product to the total number of electrons passed through the electrode during electrolysis. Assuming that the formation of one molecule of the product requires n electrons, the F.E. for urea (F.E._urea_) is calculated as follows.

3 F.E.(%)=(n×F×m)/Q×100

*n* is the number of electrons transferred per product molecule, *n* = 16 for urea formation (CO_2_ + 2NO_3_^–^ + 18H^+^ + 16e^–^ → CO­(NH_2_)_2_ + 7H_2_O), *F* is the Faraday constant (96,485 C/mol), *m* is the moles of urea produced, and *Q* is the total electric charge.

### Quantitative Measurements of Nitrite (NO_2_^–^)

The Griess assay, combined with UV–Vis spectrophotometry, was typically used to determine the resulting [NO_2_^–^].^21^ It involves a two-step chemical reaction, in which NO_2_^–^ reacts with 4-aminobenzenesulfonamide and N-(1-naphthyl)­ethylenediamine to form a pink-colored azo compound (Figure S2a). In brief, 0.5 mL of 4-aminobenzenesulfonamide solution (10 g L^–1^ in 10 wt % HCl) was added to 1.0 mL of catholyte, followed by 0.5 mL of N-(1-naphthyl) ethylenediamine dihydrochloride solution (1 g L^–1^). The mixed solution was left undisturbed for 10 min at room temperature, and the absorbance was recorded at λ = 540 nm (Figure S2b). A standard calibration curve for nitrite quantification is shown in Figure S2c.

### Quantitative Measurements of Ammonia (NH_3_)

Ammonia concentration was measured using the indophenol blue method (Figure S3a) with UV–Vis spectrophotometry.^21^ In brief, 1.0 mL of catholyte was sequentially mixed with 1.0 mL of 1 M NaOH solution containing 5 wt % salicylic acid and 5 wt % sodium citrate, followed by 0.5 mL of 0.05 M sodium hypochlorite and 0.2 mL of 1 wt % sodium nitroferricyanide. The mixture was kept in the dark for 1 h at room temperature before measurement. The absorbance was recorded at 655 nm (Figure S3b), and the calibration curve is shown in Figure S3c.

### Quantitative Measurements of Urea

Urea concentration was determined using the urease hydrolysis method (Figure S4). Specifically, 0.1 mL of urease solution (5 mg/mL) was added to 1.0 mL of catholyte and incubated at 37 °C for 40 min to decompose urea into CO_2_ and two NH_3_ molecules. The indophenol blue method was then used to determine the total ammonia content (*C*_NH3_) after urease treatment. Meanwhile, the NH_3_ concentration in the urea electrolyte without urease (*C*_0_) was also quantified by the indophenol blue method. The urea concentration (*C*_urea_) in the electrolyte and the urea yield rate normalized by the catalyst weight (*Y*_catal_) were calculated using the following equations

4 $$C_{urea}=\left(C_{NH3}-C_{0}\right)/2$$

5 $$Y_{catal}\left(\mu molh^{-1}{mg}^{-1}\right)=\left(C_{urea}\times V\right)/\left(t\times W_{catal}\right)$$

where *C*_urea_ (μmol L^–1^) is the measured urea concentration, *V* (*L*) is the volume of the electrolyte, *t* (*h*) is the reduction time, and *W*_catal_ is the catalyst weight (mg).

### In-Situ Synchrotron X-ray Absorption Spectroscopy (SXAS)

Bi L_3_- and Pd K-edge SXAS were collected at TPS BL44A (NSRRC, Hsinchu) using hard X-rays. In brief, the Bi L_3_ edge (13,419 eV) was measured in transmission mode with a Bi metal foil for simultaneous energy calibration, and the monochromator energy resolution was 0.6 eV at 13,419 eV. The Pd K edge (24,350 eV) was acquired in fluorescence mode using a Pd foil as a reference, with a resolution of 1.2 eV at 24,350 eV. All spectra were processed using Demeter (Athena/Artemis) for data processing and EXAFS fitting. Each scan was energy-aligned to the corresponding metal foil *E*_0_ (first-derivative maximum). EXAFS was Fourier transformed over *k* = 3–11.5 Å^–1^ with k^3^-weighting and a Hanning window (dk = 1.0 Å^–1^). Fitting was performed in the range *R* = 1.0–3.5 Å. Unless otherwise noted, the passive amplitude reduction factor was *S*_0_^2^ = 0.70 (determined from Bi and Pd foils). Fitting parameters follow standard notation, including *N* (coordination number), *R* (absorber–scatterer distance), and σ^2^ (Debye–Waller factor). Representative uncertainties are *N*: ± 20%, *R*: ± 1%, and σ^2^: ± 20%. The *R*-factor is reported as the goodness-of-fit metric. In-situ SXAS was conducted in a custom PTFE cell with a Kapton window using a conventional three-electrode configuration (CP working electrode, Pt wire counter electrode, and SCE reference electrode), and all potentials were converted to values vs RHE. The electrolyte was CO_2_-saturated 0.1 M KNO_3_ + 0.1 M KHCO_3_ (pH = 6.80 ± 0.03). After immersion (defined as precatalyst: BiOCl-OCP and Pd-BiOCl-OCP), potentials were stepped from 0 to −0.50 V vs RHE. The measurements were carried out under ambient pressure without electrode rotation, and a constant CO_2_ flow of 20 mL min^–1^ was maintained throughout the measurements. The cell was stabilized for 10 min at each step before data acquisition. All other acquisition parameters followed those used in the ex-situ measurements.

### In-Situ Synchrotron Powder X-ray Diffraction Measurements

SPXRD patterns were collected at NSRRC TLS BL01C2 (Hsinchu, Taiwan) using a monochromatic X-ray beam of 20 keV (λ = 0.61992 Å). The diffraction patterns were recorded with a MAR345 detector. Geometry and sample position offset corrections were calibrated using the NIST LaB_6_ (660C) standard material. One-dimensional XRD profiles were integrated from selected fan-like areas of the symmetrical two-dimensional powder rings using the GSAS-II program. In-situ SPXRD measurements were carried out in a custom PTFE cell with a Kapton window, employing a conventional three-electrode configuration (carbon paper working electrode, Pt wire counter, and SCE reference), and all potentials were converted to values vs RHE. The electrolyte was CO_2_-saturated 0.1 M KNO_3_ + 0.1 M KHCO_3_ (pH = 6.80 ± 0.03). After immersion (precatalyst states: BiOCl-OCP and Pd-BiOCl-OCP), potentials were stepped from 0 to −0.50 V vs RHE to reach urea electrosynthesis conditions. The measurements were carried out under ambient pressure without electrode rotation, and a constant CO_2_ flow of 20 mL min^–1^ was maintained throughout the measurements. The cell was stabilized for 10 min at each step before data acquisition. Other acquisition and processing parameters followed those described in the SXAS section.

## Results and Discussion

### Morphology and Composition Characterizations

BiOCl and Pd-BiOCl nanosheets were synthesized hydrothermally at a mild temperature (160 °C) using D-sorbitol as a shape-directing reagent. Figure 2a,g show SEM images of BiOCl and Pd-BiOCl nanocrystals. The BiOCl nanosheets exhibit large, well-defined rectangular shapes, whereas the Pd-BiOCl nanosheets appear smaller with rounded rectangular contours. Figure S5 presents their size-distribution histograms, where the long edges of BiOCl and Pd-BiOCl nanosheets are 452.3 ± 151.8 nm and 72.8 ± 16.7 nm, respectively. Figures S6a,d display AFM images of horizontally oriented BiOCl and Pd-BiOCl nanosheets with their rectangular faces facing upward. The AFM topographs (Figure S6b,e) show that the thicknesses are 27.0 ± 4.9 nm for the line-scanned BiOCl nanosheet in Figure S6a,b and 12.3 ± 0.8 nm for the line-scanned Pd-BiOCl nanosheet in Figure S6d,e, corresponding to approximately 35 and 17 stacked unit cells, respectively (*c* = 0.737 nm).^29^ The accuracy of the AFM measurements was further confirmed by SEM images, where the side thickness of a standing BiOCl nanosheet was 25.8 nm (Figure S6c), and that of a standing Pd-BiOCl nanosheet was 12.5 nm (Figure S6f).

**Figure 2:**
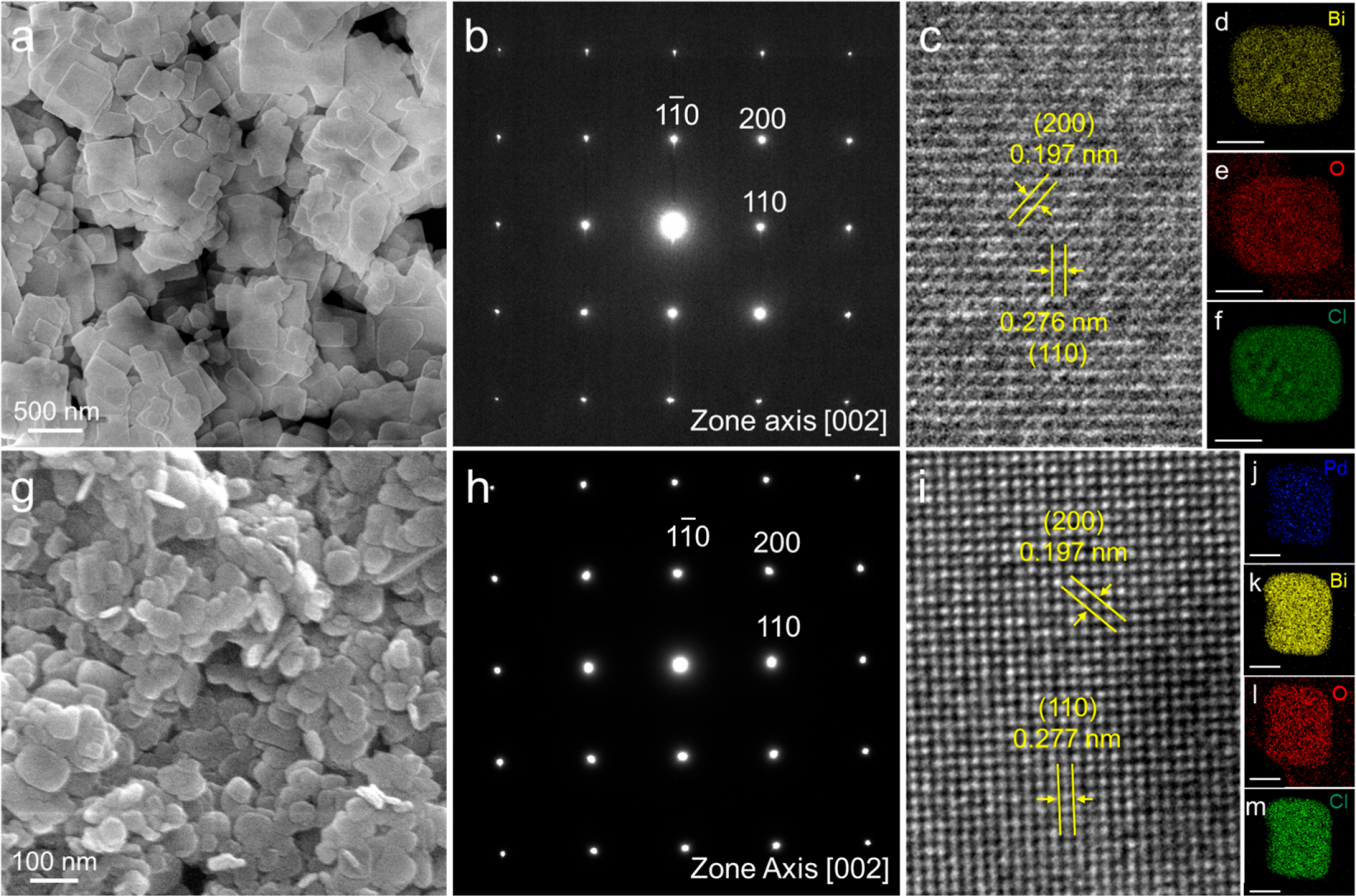
(a) SEM image, (b) SAED pattern, (c) HRTEM image, and (d–f) corresponding elemental maps for BiOCl nanosheets. (g) SEM image, (h) SAED pattern, (i) HRTEM image, and (j–m) elemental maps for Pd/BiOCl nanosheets. The colors of blue, yellow, red, and green maps represent the maps of palladium (Pd), bismuth (Bi), oxygen (O), and chlorine (Cl), respectively. The scale bar in the elemental maps corresponds to 50 nm.

Figure 2b,h show the selected-area electron diffraction (SAED) patterns obtained along the [002] zone axis, normal to the rectangular faces of single BiOCl and Pd-BiOCl nanosheets. There is no significant difference between the two patterns, indicating that the nanosheets are single-crystalline and that the incorporated Pd species do not measurably distort the BiOCl crystal lattice. The measured d-spacing values of 0.197 and 0.276 nm for the single BiOCl nanosheet (Figure 2c) correspond to the (200) and (110) crystal planes, respectively. In contrast, those of 0.197 and 0.277 nm correspond to the (200) and (110) planes of the single Pd-BiOCl nanosheet (Figure 2i). Figure 2d–f present elemental maps of a BiOCl nanosheet obtained by HAADF-STEM-EDS. The homogeneous distributions of Bi, O, and Cl confirm the BiOCl composition. Similarly, the elemental maps of Pd, Bi, O, and Cl in a Pd-BiOCl nanosheet (Figure 2j–m) also show uniform distributions, suggesting that Pd species are likely intercalated within the BiOCl nanosheets in a poorly crystalline form, consistent with the SAED observations.

### Crystal Structure Analyses

After confirming the compositions of BiOCl and Pd-BiOCl nanosheets by HAADF-STEM-EDS, their crystal structures were further examined by SPXRD. Figure 3a shows SPXRD patterns indexing both BiOCl and Pd-BiOCl nanosheets to a tetragonal crystal structure, with diffraction peaks at 2θ = 4.81°, 10.33°, 12.94°, and 13.31°, corresponding to the (001), (101), (110), and (102) crystal planes. Reference patterns of BiOCl (ICSD-195115), Bi (ICSD-64703), and Pd (ICSD-40804) were also included to validate the pure BiOCl structure. Figure 3b presents a side-view scheme of the BiOCl unit cell, projected along the (110) and (111) directions. No FCC-Pd^0^ reflections (expected at 2θ = 15.88°, 18.35°, and 26.04°) were observed in the SPXRD pattern of Pd-BiOCl nanosheets. However, Pd species were detected by inductively coupled plasma mass spectrometry (ICP-MS), with a quantified Pd content of 1.89 wt % (Table S1). These results suggest that the Pd species exist either as ions or as highly dispersed subnm clusters. The enlarged (001) diffraction peak of Pd–BiOCl shows notable broadening and a systematic shift toward lower 2θ values relative to pristine BiOCl, indicative of reduced coherent domain size along the *c*-axis and interlayer expansion upon Pd intercalation, as shown in Figure S8.

**Figure 3:**
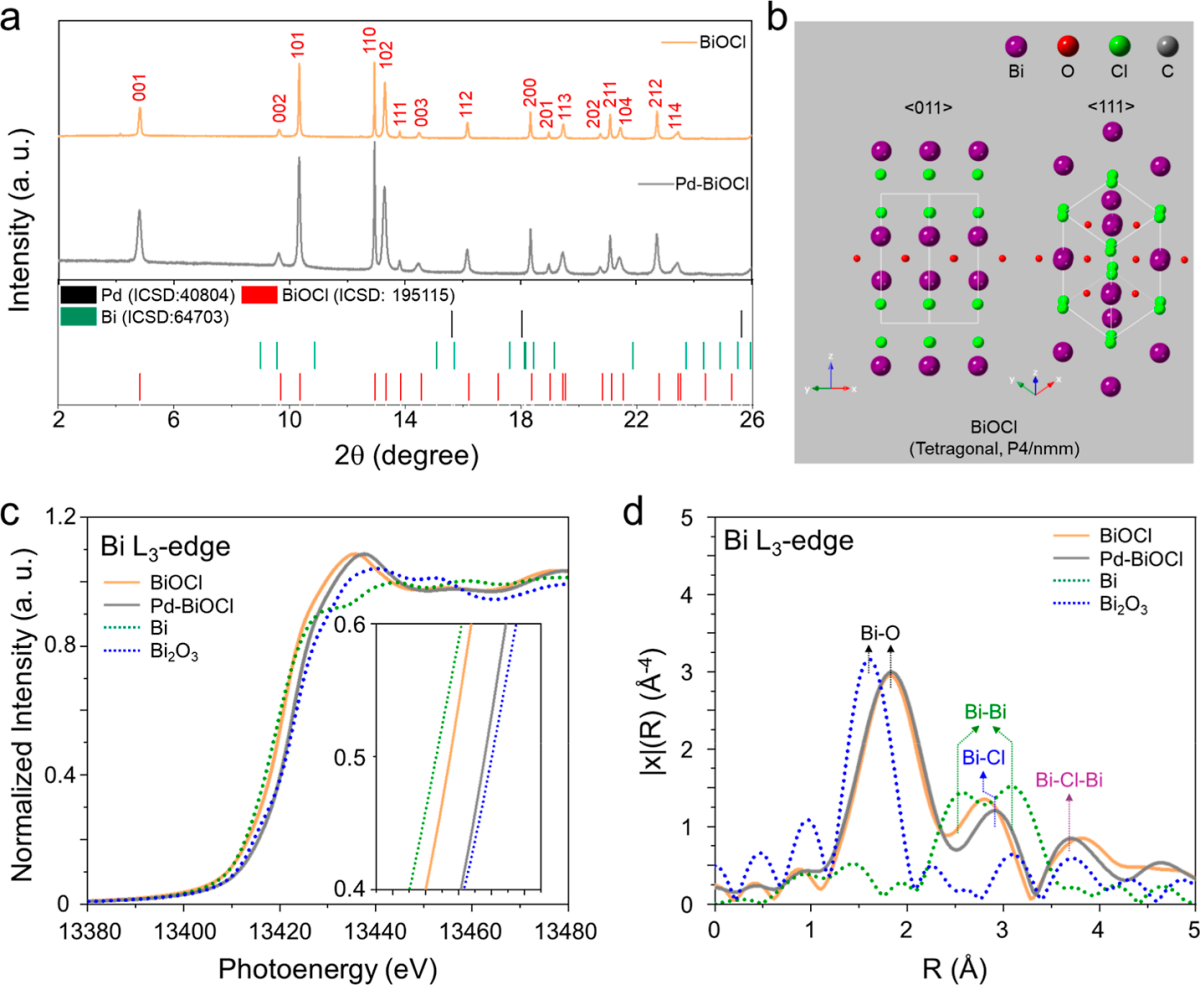
(a) SPXRD patterns of BiOCl and Pd-BiOCl nanosheets, along with the references of BiOCl (ICSD 195115), Bi (ICSD 64703), and Pd (ICSD 40804). (b) Side-view schemes of a BiOCl unit cell along <011> and <111> directions. (c) Bi L_3_-edge XANES and (d) k^3^-weighted FT-EXAFS (phase-uncorrected) of BiOCl (orange solid), Pd-BiOCl (gray solid), Bi (green dashed), and Bi_2_O_3_ (blue dashed). Peaks in (d) are phase-uncorrected apparent distances, thus shorter than actual bond lengths.

### Electronic Structure Analyses

Figure 3c presents the Bi L_3_-edge X-ray absorption near-edge structures (XANES) of BiOCl and Pd-BiOCl nanosheets. The maximal white-line energies are 13,437.23 eV for BiOCl and 13,438.10 eV for Pd-BiOCl, consistent with the XANES feature of Bi^3+^ (Bi_2_O_3_) but not Bi^0^ (Bi foil). The white-line energy, which correlates with the unoccupied 6p orbital and local coordination (and thus with the average Bi–O distance), shifts to higher energy upon Pd intercalation.^26,30^ Notably, the edge energy *E*_0_ (defined at 0.5 normalized intensity) increases from 13,421.30 eV for BiOCl to 13,422.10 eV for Pd-BiOCl. In contrast, the Pd K-edge XANES of Pd-BiOCl appears at a slightly lower energy than Pd^0^ (Figure S9a). These results reveal not only charge transfer from Bi to Pd but also the actual presence of tiny Pd subnm clusters with extremely poor crystallinity. Therefore, the Pd subnm clusters are likely intercalated between the layers of the BiOCl unit cells, as demonstrated in previous work.^26^

To investigate the local coordination of BiOCl and Pd-BiOCl nanosheets, k^3^-weighted, phase-uncorrected Fourier-transform EXAFS (FT-EXAFS) spectra were computed using a Hanning window over *k* = 2.7–10.3 Å^–1^ (Figure 3d). The Bi coordination environment in BiOCl is consistent with the tetragonal crystal structure determined by SPXRD.^31^ The first-shell Bi–O peak appears near ∼1.8 Å in the R-space spectra (phase-uncorrected) for both BiOCl and Pd–BiOCl. Additional features assignable to Bi–Cl are observed at ∼2.83 Å for BiOCl and ∼2.85 Å for Pd–BiOCl (both phase-uncorrected). No features attributable to metallic Bi, such as Bi–Bi contributions (∼2.58 Å and ∼3.18 Å, phase-uncorrected), are observed in either sample. Quantitative FT-EXAFS fitting (Table S2) resolves the Bi–O and Bi–Cl paths for BiOCl and Pd-BiOCl nanosheets, yielding bond lengths and coordination numbers (*CN*_s_). Representative fits are shown in Figure S7. For BiOCl, the fitted bond lengths are Bi–O = 2.27 Å, Bi–Cl = 3.04 Å, and Bi–O–Bi = 3.74 Å; for Pd-BiOCl, Bi–O = 2.28 Å, Bi–Cl = 3.05 Å, and Bi–O–Bi = 3.75 Å. These values are statistically indistinguishable within fitting uncertainties. The corresponding coordination numbers are CN­(Bi–O) = 4.02 and CN­(Bi–Cl) = 4.11 for BiOCl, and CN­(Bi–O) = 3.65 and CN­(Bi–Cl) = 3.77 for Pd-BiOCl. In contrast, the higher-shell Bi–Bi coordination number decreases from 6.50 (BiOCl) to 6.03 (Pd-BiOCl), indicating that Pd intercalation weakens Bi–Bi bonding. Figure S9b shows the Pd K-edge FT-EXAFS of Pd-BiOCl nanosheets with Pd foil as a reference. Since the Pd K-edge XANES resembles metallic Pd^0^ (Figure S9a), the FT-EXAFS reveals a Pd–Pd contribution. Its reduced amplitude relative to Pd foil, together with the fitted Pd–Pd CN = 6.4.^32^ In addition, a weak Pd–Bi contribution (CN ≈ 1.1 from the Pd center and CN ≈ 1.7 for Bi–Pd from the Bi center) supports the existence of interfacial Pd–Bi coordination, rationalizing the reduction in Bi–Bi coordination number upon Pd intercalation.

To elucidate the influence of Pd doping on the electronic structure of BiOCl nanosheets, high-resolution X-ray photoelectron spectroscopy (HR-XPS) was performed. The results reveal a distinct electronic reconfiguration in the Pd-BiOCl samples compared to the pristine BiOCl. As shown in Figure S10a, the binding energy (BE) of Bi 4f_7/2_ exhibits a slight downward shift from 158.92 to 158.79 eV (ΔBE = −0.13 eV), suggesting a marginal enrichment of electron density at the Bi sites. On the other hand, more pronounced changes are observed within the anionic frameworks. The lattice oxygen in O 1s spectra (Figure S10b) manifests a downshift from 529.70 to 529.53 eV. These collective shifts signify a substantial electron flow from the Pd-perturbed environment toward the BiOCl lattice. The Cl 2p spectra (Figure S10c) show a shift toward lower binding energy (from 199.24 to 199.12 eV), indicating an increased electron density around Cl atoms upon Pd incorporation.

The chemical state of the dopant was further examined via Pd 3d spectra (Figure S10d). The Pd 3d_5/2_ peak at 339.07 eV exhibits a significant upshift compared to Pd^0^.^26^ This observation, coupled with the Pd^0^ signature in SXAS, suggests that the intercalated Pd exists as subnanometer clusters. The pronounced binding energy shift originates from the combination of quantum size effects and strong electronic metal–support interaction (EMSI), where the electronegative Cl-terminated layers induce a localized electron deficiency at the interface of Pd and BiOCl.^33,34^ While Pd K-edge SXAS confirms that the Pd exists in a reduced metallic state characterized by dominant Pd–Pd coordination, which can be defined as a nano intercalator.^35,36^ The apparent discrepancy between the HR-XPS and SXAS results provides critical insight into the electronic metal support interaction within the Pd-BiOCl system. Pd K edge SXAS confirms that Pd predominantly exists in a reduced metallic state characterized by dominant Pd–Pd coordination, consistent with a nanointercalated metallic cluster structure. In contrast, HR-XPS selectively probes the interfacial electronic environment and reveals pronounced charge redistribution at the heterojunction. The synchronous downshift in the binding energies of Bi, O, and Cl indicates that metallic Pd subnm clusters function as electron donors, injecting electron density into the BiOCl framework. The high binding energy Pd 3d component at 339.07 eV corresponds to interfacial Pd atoms that become electron-deficient due to strong electronic coupling with the electronegative Cl layers.^34^ These results demonstrate a localized electronic asymmetry within the Pd species. HR-XPS analysis identifies dominant Pd–Cl interactions with negligible Pd–O contributions, suggesting that the primary interfacial charge polarization originates from coupling with the Cl-terminated surface. However, EXAFS fitting requires inclusion of a Pd–O coordination shell in addition to the metallic Pd–Pd coordination pathway with a coordination number of 6.4. This observation supports a subsurface anchoring model in which Pd subnm clusters are embedded within the BiOCl lattice and remain in geometric proximity to internal oxygen layers, while the primary electronic polarization occurs through interfacial Pd–Cl bonding. XANES further confirms preservation of a coherent metallic Pd core, ensuring efficient electron transport during catalytic cycles. This structural configuration, consisting of a metallic core and a highly polarized electron donating interface, generates a spatial electronic gradient that optimizes the surface electronic structure and underlies the enhanced catalytic performance of Pd-BiOCl. According to HR-XPS measurements, the Pd subnm cluster exhibits a prominent Pd^δ+^ component at the interface with the Cl layers, indicating substantial electron transfer from Pd clusters to electronegative chlorine species. In contrast, SXAS data indicate that the interior region of the Pd subnm cluster remains predominantly metallic, with Pd in a metallic state. This, characterized by a cationic interface and a metallic core, highlights site-specific electronic modulation within the intercalated nanostructure.^34^

### Electrocatalytic Synthesis of Urea

Electrosynthesis of urea was evaluated in a gastight H-cell (Figure S1) under ambient conditions. An aqueous solution containing 0.10 M KHCO_3_ and 0.10 M KNO_3_ was used as the electrolyte. Linear-sweep voltammograms showed higher currents when the electrolyte was saturated with CO_2_ than with Ar (Figure 4a), indicating CO_2_/NO_3_^–^ coreduction leading to C–N coupling. Notably, Pd-BiOCl-WE exhibited the highest geometric current across the examined potential range among the tested electrocatalysts. In Figure 4b, Nyquist plots from electrochemical impedance spectroscopy (EIS) illustrate the impedance characteristics of BiOCl-WE (orange), Pd-BiOCl-WE (gray), and Pd/C (purple) at −0.3 V (vs RHE). The inset in Figure 4b shows the equivalent circuit model used to fit the semicircles, with detailed parameters listed in Table S3. The circuit includes *R*_s_, representing the overall internal resistance (contributions from electrode and electrolyte), and *R*_ct_, the charge-transfer resistance at the electrode–electrolyte interface. The constant phase element (CPE) consists of CPE_T1_, representing nonideal interfacial capacitance, and its exponent CPE_P1_, which ranges from 0 to 1. The fitting results in Table S3 show that the *R*_s_ values for BiOCl-WE, Pd-BiOCl-WE, and Pd/C-WE are similar: 30.42 Ω, 31.38 Ω, and 29.17 Ω, respectively. *R*_ct_ decreases from 225.9 Ω for BiOCl-WE to 136.9 Ω for Pd-BiOCl-WE, a reduction of about 46%, indicating that Pd intercalation accelerates interfacial charge transfer under applied potentials. However, Pd/C-WE still shows the lowest *R*_ct_ of 79.97 Ω, reflecting Pd’s superior conductivity. To estimate the electrochemically active surface areas (ECSA), the electrochemical double-layer capacitances (*C*_dl_) of all working electrodes were determined from *I*–*E* plots recorded within a nonfaradaic window (Figure S11). Cyclic voltammograms (CVs) were collected at the open-circuit potential (OCP) ± 0.05 V (vs RHE) at sweep rates (r) of 20, 40, 60, 80, and 100 mV/s for BiOCl-WE (Figure S11a), Pd-BiOCl-WE (Figure S11b), and Pd/C-WE (Figure S11c). Figure 4c shows Δ*I*_OCP_/2 vs sweep rate (*r*), with linear correlations enabling estimation of *C*_dl_ values. Specifically, *C*_dl_ values are 0.010 mF for BiOCl-WE, 0.054 mF for Pd-BiOCl-WE, and 0.062 mF for Pd/C-WE. Hence, the ECSA values, calculated as *C*_dl_/Cs, are 0.25 cm^2^ for BiOCl-WE, 1.35 cm^2^ for Pd-BiOCl-WE, and 1.57 cm^2^ for Pd/C-WE. These *C*_dl_ and ECSA values are summarized in Figure 4d and Table S4. While EIS highlights the nonideal capacitive behavior (CPE) associated with charge transfer at reactive potentials, CV-based *C*_dl_ measurements provide a standardized estimate of the electrochemically active surface area (ECSA). Both techniques consistently show that Pd intercalation significantly increases the density of accessible sites and lowers the interfacial charge-transfer resistance, collectively boosting the urea yield rate.^37^

**Figure 4:**
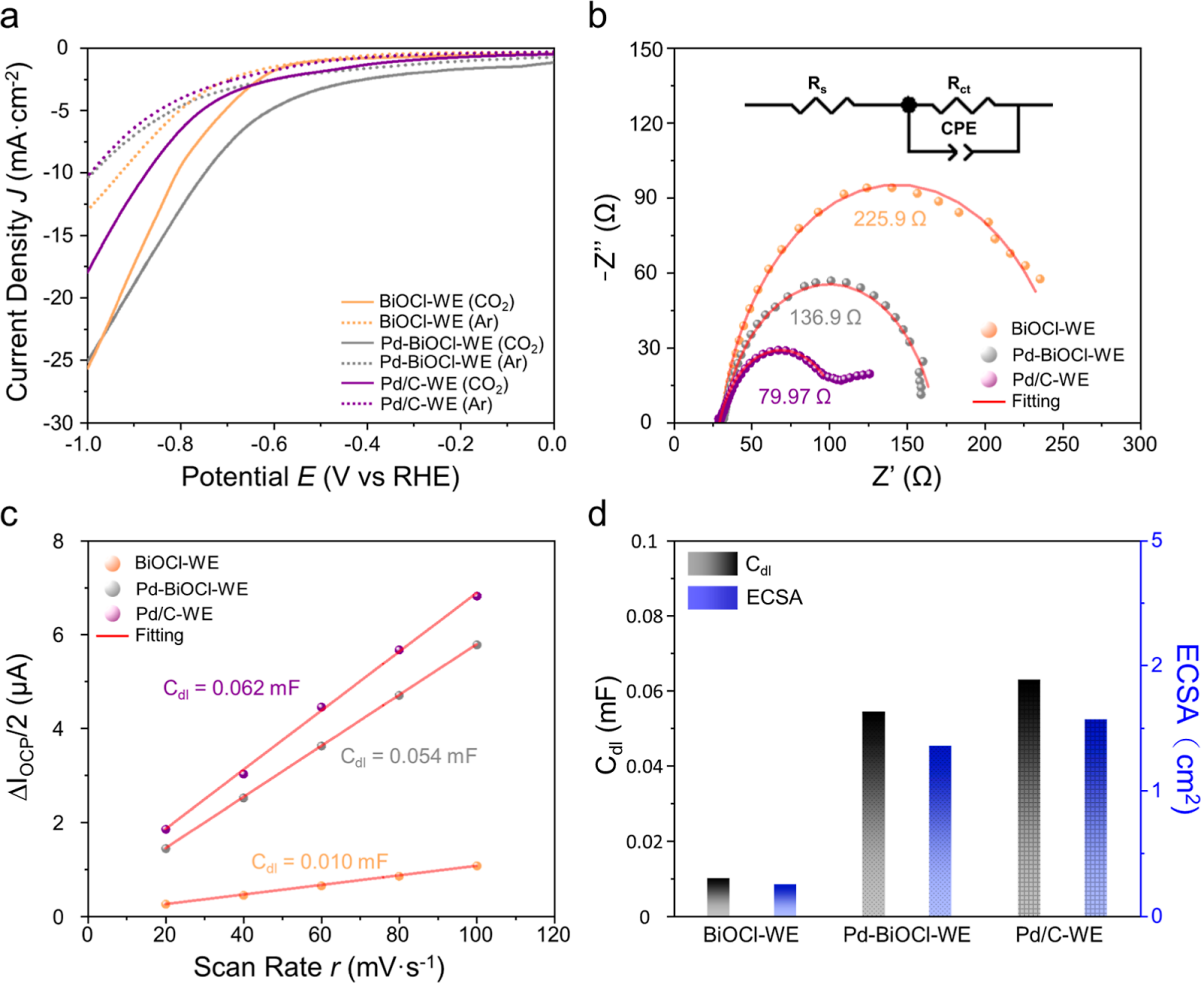
(a) LSVs of BiOCl-WE (orange), Pd–BiOCl-WE (gray), and Pd/C-WE (purple) in CO_2_-saturated (solid lines) and Ar-saturated (dashed lines) 0.1 M KNO_3_ + 0.1 M KHCO_3_, recorded without *iR* compensation. (b) EIS Nyquist plots under CO_2_ purging at *E* = −0.3 V vs RHE with a 5 mV perturbation over 100 kHz to 0.1 Hz. (c) The plots of Δ*I*_OCP_/2 vs *r* acquired at a nonfaradaic potential near OCP for BiOCl-WE, Pd–BiOCl-WE, and Pd/C-WE. (d) The *C*_dl_ and ECSA for BiOCl-WE, Pd–BiOCl-WE, and Pd/C-WE.

Potential-dependent urea electrosynthesis was performed for 1 h at applied potentials from −0.2 to −0.6 V (vs RHE) in a CO_2_-saturated electrolyte containing 0.1 M KHCO_3_ and 0.1 M KNO_3_. Faradaic efficiencies (F.E.) were calculated according to eq 3. F.E. profiles for BiOCl-WE, Pd-BiOCl-WE, and Pd/C-WE are shown in Figure 5a–c, with all values summarized in Table S5. At −0.3 V, F.E.(urea) reached 6.99 ± 1.62% for BiOCl-WE and 12.43 ± 1.02% for Pd–BiOCl-WE, whereas no detectable urea was observed for Pd/C-WE. In addition, BiOCl-based working electrodes significantly suppressed the hydrogen evolution reaction (HER), yielding lower F.E.(H_2_) values from −0.2 to −0.6 V compared with Pd/C-WE. For example, at −0.3 V, F.E.(H_2_) was 20.67 ± 1.18% for Pd/C-WE, but only 12.65 ± 0.79% for BiOCl-WE and 9.28 ± 1.23% for Pd–BiOCl-WE. These results highlight the key role of BiOCl in directing electrons more efficiently toward C–N coupling rather than water reduction. Meanwhile, because Pd/C-WE exhibits both larger ECSA and lower charge-transfer resistance (*R*_ct_), the results suggest that high surface area and facile charge transfer may favor HER over C–N coupling.

**Figure 5:**
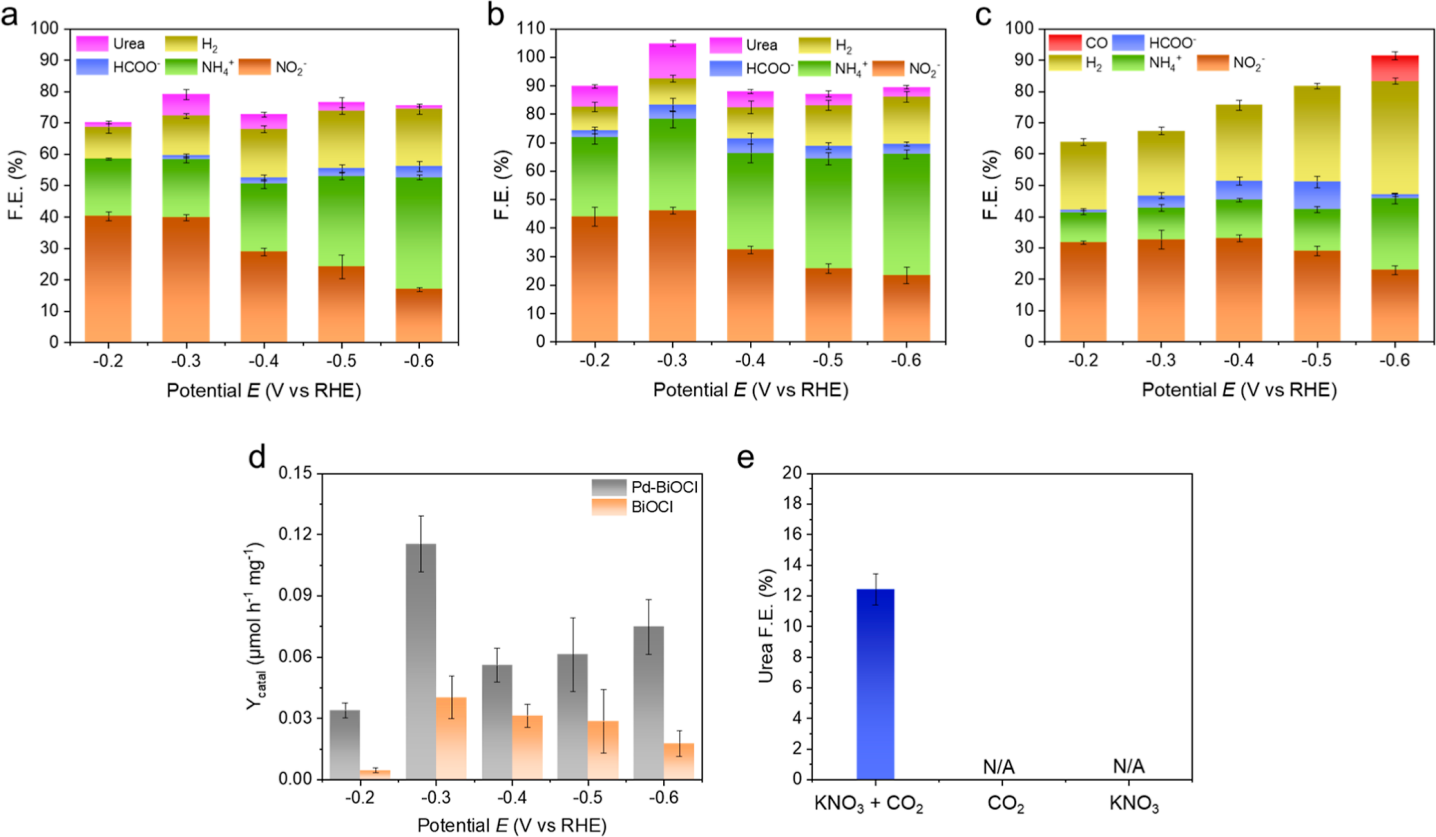
Faradaic efficiencies (F.E.) of products formed from the electrosynthesis of urea over (a) BiOCl-WE, (b) Pd-BiOCl-WE, and (c) Pd/C-WE. (d) Yield rates normalized by catalyst weights obtained with Pd-BiOCl-WE (gray) and BiOCl-WE (orange). (e) The urea F.E. obtained over Pd-BiOCl-WE at −0.3 V (vs RHE) in different electrolytes.

### Quantitative Determination of Urea

To quantify urea productivity, the yield rate normalized by catalyst mass (*Y*_catal_) was calculated using eq 5 and summarized in Figure 5d and Table S6. Both Pd-BiOCl-WE and BiOCl-WE exhibit a volcano-type dependence of *Y*_catal_ on applied potential due to optimized kinetics of NO_3_RR and CO_2_RR. The highest *Y*_catal_ is observed for Pd-BiOCl-WE, 0.1160 ± 0.0136 μmol h^–1^ mg^–1^, which is 2.88 times higher than that of BiOCl-WE (0.0403 ± 0.0012 μmol h^–1^ mg^–1^) with the same nanosheet loading. To examine the influence of the electrolyte, three control experiments using KNO_3_/CO_2_, only CO_2_, and only KNO_3_ were performed at −0.3 V over Pd-BiOCl-WE (Figure 5e). The results indicate that both NO_3_^–^ and CO_2_ are essential, as their absence leads to negligible urea production. Urea production over Pd-BiOCl-WE peaks at −0.3 V, where carbon- and nitrogen-derived intermediates coexist while surface *H is suppressed. These observations support a coreduction pathway involving CO_2_RR and NO_3_RR. Therefore, the catalytic performance was further evaluated for intrinsic CO_2_RR and NO_3_RR. Figure S12, Tables S7 and S8 summarize the F.E. results of CO_2_RR and NO_3_RR over BiOCl-WE, Pd-BiOCl-WE, and Pd/C-WE. For CO_2_RR (Figures S12a–c), Pd-BiOCl-WE exhibits higher F.E.(formate) values than BiOCl-WE across −0.2 to −0.6 V, consistent with previous work.^26^ This indicates that intercalated Pd subnm clusters improve the conductivity (lower *R*_ct_) of BiOCl nanosheets. Moreover, Pd/C-WE exhibits much higher F.E.(H_2_) from water reduction and CO from CO_2_RR, whereas Pd-BiOCl-WE does not. This difference suggests that the intercalated Pd subnm clusters do not directly participate in CO_2_RR.^38^ For NO_3_RR, all working electrodes show F.E.(NH_4_^+^) increasing with more negative potentials. However, the F.E.(NH_4_^+^) values for BiOCl-WE and Pd–BiOCl-WE are higher than those for Pd/C-WE across −0.2 to −0.6 V. These results imply that urea formation is strongly correlated with C–N coupling between the reduced intermediates from CO_2_ and NO_3_^–^, such as formate and ammonium, thereby validating the catalytic role of BiOCl in urea electrosynthesis. Figure S13 shows chronoamperometry at −0.3 V for 100 h over BiOCl-WE and Pd–BiOCl-WE. A clear increase in current density for Pd–BiOCl-WE (57.5%) is observed after 100 h, whereas BiOCl-WE shows only a 10.5% increase. These results raise an open question about the mechanism of BiOCl-catalyzed urea production and how Pd^0^ contributes to improving efficiency over time.

### Operando Study by Synchrotron X-ray Spectroscopies

To shed light on the mechanism of BiOCl-catalyzed urea electrosynthesis, real-time investigation of the generated intermediates is essential. Therefore, operando SPXRD and SXAS were performed using BiOCl-WE and Pd–BiOCl-WE for urea electrosynthesis in a CO_2_-saturated electrolyte containing 0.10 M KHCO_3_ and 0.10 M KNO_3_. Each applied potential was held for 10 min from 0 to −0.50 V. For operando SPXRD, all peaks in the patterns were indexed against reference patterns for Bi, Pd, BiOCl, and Bi_2_O_2_CO_3_ (ICSD 202767). The generation of Bi_2_O_2_CO_3_ has been observed in Hua’s work, confirming our reliable results.^39^ Figure 6a,b show sequential SPXRD patterns collected at open-circuit potential (BiOCl-OCP, Pd-BiOCl-OCP) and under the stepwise potential sequence. At OCP, the diffraction patterns of BiOCl-OCP and Pd-BiOCl-OCP align well with the reference pattern of tetragonal BiOCl, indicating that immersion in the electrolyte preserves the intact framework of BiOCl nanosheets. However, signals of Bi_2_O_2_CO_3_ (schematic view of unit cell in Figure S14) appear at onset potentials of −0.20 V for BiOCl-WE and −0.10 V for Pd–BiOCl-WE, with intensities increasing steadily up to −0.50 V. The lower onset potential for Pd–BiOCl-WE suggests that intercalated Pd^0^ promotes the anion-exchange conversion of BiOCl to Bi_2_O_2_CO_3_, attributed to interlayer expansion/anion substitution by CO_2_. On the other hand, a weak peak assignable to metallic Bi^0^ is only observed for BiOCl-WE at −0.50 V (Figure S15), clearly indicating partial reduction from Bi^3+^ to Bi^0^ on the low-conductive BiOCl nanosheets under negative potentials, owing to the absence of assistance from metallic Pd subnm clusters. From these observations, it can be inferred that during CO_2_ reduction in bicarbonate electrolytes, CO_2_ near the BiOCl electrode equilibrates with HCO_3_^–^/CO_3_^2–^. These CO_3_^2–^ anions diffuse into the layered [Cl–Bi–O–Bi–Cl] framework and replace interlayer Cl^–^ to form CO_3_^2–^ dangling bonds, yielding a Bi_2_O_2_CO_3_ subsurface layer. Interlayer CO_3_^2–^ insertion is enhanced by Pd intercalation, which increases the collision frequency with nitrogen-containing species (NO_2_^–^ and NH_4_^+^), leading to improved urea production at lower overpotentials.

**Figure 6:**
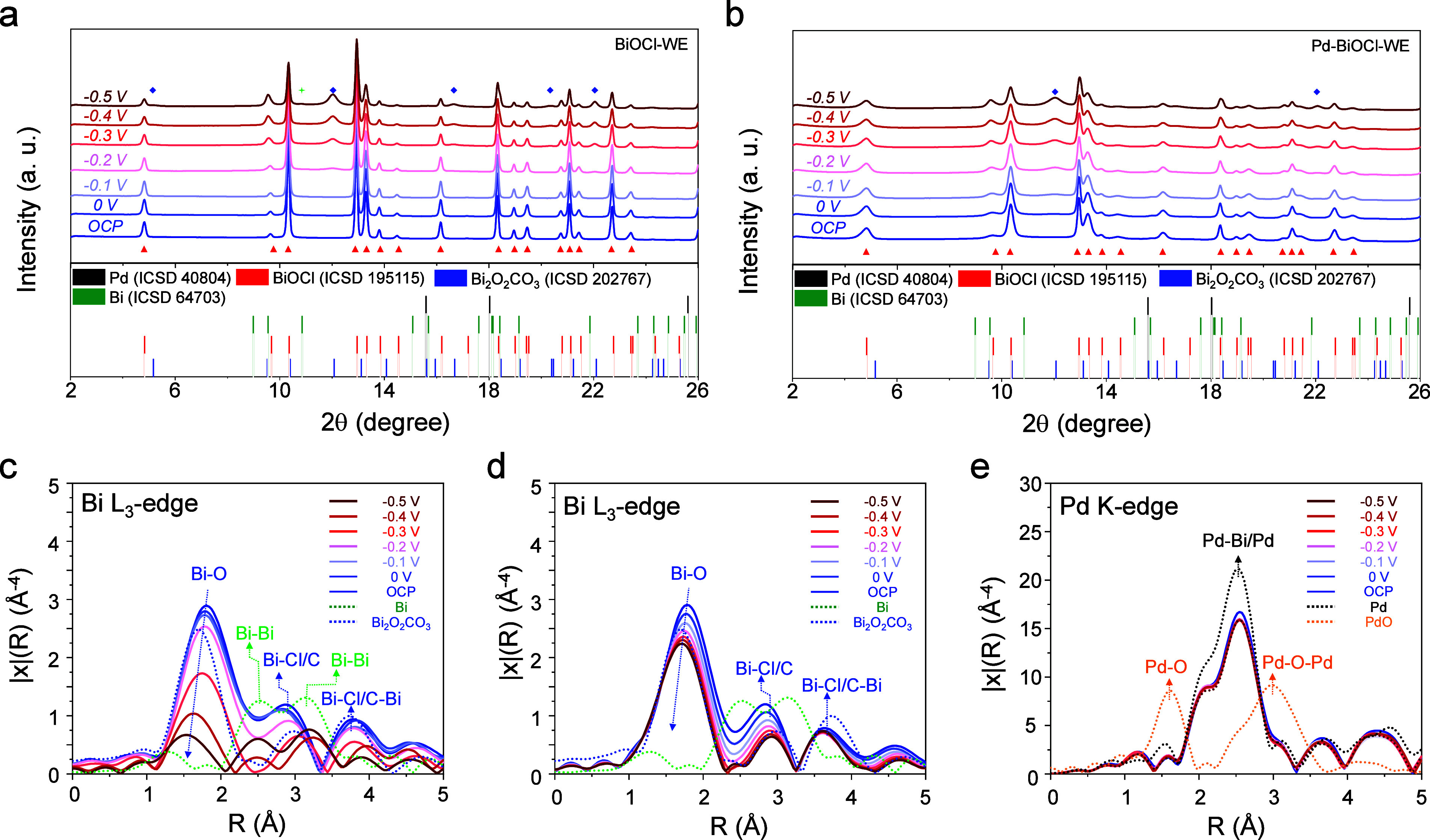
Operando (a,b) SPXRD and (c–e) SXAS spectra in the process of urea electrosynthesis. Potential-dependent SPXRD for (a) BiOCl-WE and (b) Pd–BiOCl-WE recorded from 0 to −0.5 V, in which every potential was held for 10 min k^3^-weighted magnitude FT-EXAFS at the Bi L_3_-edge for (c) BiOCl-WE and (d) Pd–BiOCl-WE, and the (e) Pd K-edge for Pd–BiOCl-WE obtained under the same conditions to those for SPXRD.

Figure 6c,d present the k^3^-weighted Fourier transform magnitudes of Bi L_3_-edge EXAFS for BiOCl-OCP and Pd-BiOCl-OCP. These spectra were obtained by immersing dry BiOCl-WE and Pd–BiOCl-WE into a CO_2_-saturated electrolyte without applying potential. Their EXAFS spectra are consistent with those of BiOCl-WE and Pd–BiOCl–WE, indicating no significant electrolyte-induced damage to the BiOCl crystal structure. Upon stepping the potential from 0 to −0.50 V, the EXAFS spectrum of BiOCl-WE exhibits potential-dependent changes. First, the first-shell Bi–O bond shifts to a shorter distance, while the Bi–Cl contribution becomes assignable to Bi–C, reflecting the formation of Bi_2_O_2_CO_3_, also confirmed by SPXRD at −0.20 V. Second, between −0.30 and −0.50 V, a new contribution attributable to Bi–Bi scattering emerges, indicating partial reduction of Bi^3+^ in BiOCl toward metallic Bi. In contrast, Pd–BiOCl-WE exhibits an earlier Bi–Cl to Bi–C transformation, starting at −0.10 V, which verifies that Pd-intercalation facilitates anion exchange toward Bi_2_O_2_CO_3_. From −0.10 to −0.50 V, the Bicentered local environment remains Bi_2_O_2_CO_3_-like, with no resolvable Bi–Bi contribution detected within SXAS sensitivity, again suggesting that Pd intercalation stabilizes the BiOCl/Bi_2_O_2_CO_3_ framework and retards metallic Bi^0^ formation. Pd K-edge FT-EXAFS shows no discernible change during urea electrosynthesis, indicating that Pd subnm clusters remain metallic (Figure 6e). Considering all SXAS results, Pd intercalation not only strengthens the structural robustness of Pd–BiOCl but also enhances conductivity, thereby improving urea electrosynthesis activity. Notably, Pd–BiOCl-WE exhibits higher urea Faradaic efficiency than BiOCl-WE, attributable to two synergistic effects: (i) Pd facilitates charge transfer within the BiOCl matrix, promoting CO_2_/NO_3_^–^ coreduction, and (ii) Pd intercalation mitigates the over-reduction of Bi species, enabling partial conversion of BiOCl to Bi_2_O_2_CO_3_, while no clear evidence of Bi^0^ formation is observed under the investigated conditions, thereby preserving the layered framework. In contrast, BiOCl-WE without Pd undergoes extensive transformation to Bi^0^, compromising the integrity of the BiOCl lattice and reducing catalytic selectivity. Guided by structural and electrochemical characterizations, BiOCl-based catalysts suppress the hydrogen evolution reaction (HER) and promote C–N coupling, enabling urea formation at lower negative potentials. Under CO_2_/KHCO_3_, BiOCl undergoes intercalative anion exchange, converting to Bi_2_O_2_CO_3_ starting at −0.2 V. At −0.5 V, further reduction partially yields metallic Bi^0^, accompanied by collapse of the layered framework and loss of activity/selectivity. In contrast, Pd intercalation exerts a bifunctional effect, simultaneously enhancing catalytic activity and reinforcing structural stability. The electronic metal–support interaction (EMSI) between Pd and the BiOCl lattice induces interfacial charge redistribution, thereby strengthening the intrinsic chemical bonding within the layered framework. Concurrently, the spatial confinement introduced by Pd intercalation establishes a stabilized microenvironment that kinetically limits excessive CO_3_^2–^ incorporation into the lattice. Operando spectroscopic analysis verifies that the intercalated Pd remains metallic under reaction conditions and operates as an efficient electron-transfer hub. The metallic Pd core promotes CO_2_/NO_3_^–^ coreduction by accelerating interfacial electron transport and furnishing active sites for C–N coupling, while suppressing the reduction pathway from Bi_2_O_2_CO_3_ to metallic Bi^0^. This Pd-mediated modulation of charge transfer preserves the integrity of the BiOCl-derived layered structure during operation and markedly enhances the Faradaic efficiency for urea production. The synergistic interplay between electronic regulation and geometric confinement effectively inhibits the unfavorable phase transition from BiOCl to Bi_2_O_2_CO_3_ and prevents subsequent deep reduction of bismuth species. Consequently, Pd-intercalated BiOCl maintains structural robustness and catalytic durability even under rigorous operando conditions.

## Conclusions

We hydrothermally synthesized BiOCl and Pd-blended BiOCl nanosheets and investigated their electrocatalytic performances in urea production, comparing them with the commercial Pd/C catalyst. After synthesis, the blended Pd precursor formed subnm clusters that were intercalated within the interlayers of the BiOCl nanosheets. Under ambient conditions, the maximum F.E.(urea) reached 6.99 ± 1.62% for BiOCl, 12.43 ± 1.02% for Pd–BiOCl, and 0% for Pd/C at −0.30 V (vs RHE). Although Pd/C produces only CO without detectable urea, this does not contradict the promotional role of Pd in the Pd–BiOCl system. In Pd/C, Pd exists as isolated metallic nanoparticles without strong electronic coupling to a catalytically active oxide lattice, and therefore primarily catalyzes CO_2_-to-CO conversion. In contrast, within Pd–BiOCl, intercalated Pd subnm clusters are electronically integrated into the BiOCl framework through strong metal–support interaction. The metallic Pd core functions as an electron-transfer hub and modifies the local electronic structure of adjacent Bi–O–Cl layers. Post/operando characterizations revealed that BiOCl partially converted to Bi_2_O_2_CO_3_ through interlayer anion exchange (Cl^–^ → CO_3_^2–^), beginning at −0.20 V (vs RHE). At −0.50 V, over-reduction occurred in BiOCl nanosheets, leading to the formation of metallic Bi nanoclusters and the collapse of the layered framework, thereby significantly reducing the urea yield rate. In contrast, intercalated Pd subnm clusters in BiOCl nanosheets confer advantages by stabilizing the layered structure, mitigating Bi over-reduction (with no clear evidence of Bi^0^ formation under the investigated conditions), lowering the charge-transfer resistance, and enhancing anion exchange at the BiOCl interlayers for C–N coupling. Our findings introduce a novel concept for increasing collision frequency between C- and N-species within nanosheet interlayers, providing a significant benchmark for catalyst design in nanocatalysis.

## Supplementary Material



## Notes

Including SEM, AFM images, FT-EXAFS spectra, size distribution, XAS spectroscopy, and Tables (PDF)

## References

[1] Chen J. G., Crooks R. M., Seefeldt L. C., Bren K. L., Bullock R. M., Darensbourg M. Y., Holland P. L., Hoffman B., Janik M. J., Jones A. K. (2018). Beyond Fossil Fuel–Driven Nitrogen Transformations. Science.

[2] Kyriakou V., Garagounis I., Vourros A., Vasileiou E., Stoukides M. (2020). An Electrochemical Haber-Bosch Process. Joule.

[3] Lim J., Fernández C. A., Lee S. W., Hatzell M. C. (2021). Ammonia and Nitric
Acid Demands for Fertilizer Use in 2050. ACS
Energy Lett..

[4] Smith C., Hill A. K., Torrente-Murciano L. (2020). Current and
Future Role of Haber–Bosch
Ammonia in a Carbon-Free Energy Landscape. Energy
Environ. Sci..

[5] Chen C., Zhu X., Wen X., Zhou Y., Zhou L., Li H., Tao L., Li Q., Du S., Liu T. (2020). Coupling
N_2_ and CO_2_ in H_2_O to Synthesize Urea
under Ambient Conditions. Nat. Chem..

[6] Jiang M., Zhu M., Wang M., He Y., Luo X., Wu C., Zhang L., Jin Z. (2023). Review on
Electrocatalytic Coreduction
of Carbon Dioxide and Nitrogenous Species for Urea Synthesis. ACS Nano.

[7] Huang Y., Yang R., Wang C., Meng N., Shi Y., Yu Y., Zhang B. (2022). Direct electrosynthesis
of urea from carbon dioxide
and nitric oxide. ACS Energy Lett..

[8] Shibata M., Yoshida K., Furuya N. (1995). Electrochemical Synthesis
of Urea
on Reduction of Carbon Dioxide with Nitrate and Nitrite Ions Using
Cu-Loaded Gas-Diffusion Electrode. J. Electroanal.
Chem..

[9] Peng X., Zeng L., Wang D., Liu Z., Li Y., Li Z., Yang B., Lei L., Dai L., Hou Y. (2023). Electrochemical
C–N Coupling of CO_2_ and Nitrogenous Small Molecules
for the Electrosynthesis of Organonitrogen Compounds. Chem. Soc. Rev..

[10] Liu J., Guo X., Frauenheim T., Gu Y., Kou L. (2024). Urea Electrosynthesis
Accelerated by Theoretical Simulations. Adv.
Funct. Mater..

[11] Li J., Li S., Zhang Y., Liu Z. Q. (2024). Principles of Designing
Electrocatalysts
to Boost C–N Coupling Reactions for Urea Synthesis. EcoEnergy.

[12] Wei X., Wen X., Liu Y., Chen C., Xie C., Wang D., Qiu M., He N., Zhou P., Chen W. (2022). Oxygen
Vacancy-Mediated Selective C–N Coupling toward Electrocatalytic
Urea Synthesis. J. Am. Chem. Soc..

[13] Lv C., Lee C., Zhong L., Liu H., Liu J., Yang L., Yan C., Yu W., Hng H. H., Qi Z. A. (2022). Defect
Engineered Electrocatalyst that Promotes High-Efficiency Urea Synthesis
under Ambient Conditions. ACS Nano.

[14] Meng N., Huang Y., Liu Y., Yu Y., Zhang B. (2021). Electrosynthesis
of Urea from Nitrite and CO_2_ over Oxygen Vacancy-Rich ZnO
Porous Nanosheets. Cell Rep. Phys. Sci..

[15] Lv L., Tan H., Kong Y., Tang B., Ji Q., Liu Y., Wang C., Zhuang Z., Wang H., Ge M. (2024). Breaking the Scaling Relationship in C–N Coupling via the
Doping Effects for Efficient Urea Electrosynthesis. Angew. Chem., Int. Ed..

[16] Meng N., Ma X., Wang C., Wang Y., Yang R., Shao J., Huang Y., Xu Y., Zhang B., Yu Y. (2022). Oxide-Derived
Core–Shell Cu@ Zn Nanowires for Urea Electrosynthesis from
Carbon Dioxide and Nitrate in Water. ACS Nano.

[17] Song X., Ma X., Chen T., Xu L., Feng J., Wu L., Jia S., Zhang L., Tan X., Wang R. (2024). Urea Synthesis
via Coelectrolysis of CO_2_ and Nitrate over Heterostructured
Cu–Bi Catalysts. J. Am. Chem. Soc..

[18] Liu S., Yin S., Wang Z., Xu Y., Li X., Wang L., Wang H. (2022). AuCu Nanofibers for Electrosynthesis
of Urea from Carbon Dioxide
and Nitrite. Cell Rep. Phys. Sci..

[19] Leverett J., Tran-Phu T., Yuwono J. A., Kumar P., Kim C., Zhai Q., Han C., Qu J., Cairney J., Simonov A. N. (2022). Tuning the Coordination Structure of Cu–N–C
Single Atom Catalysts for Simultaneous Electrochemical Reduction of
CO_2_ and NO^3–^ to Urea. Adv. Energy Mater..

[20] Li Y., Zheng S., Liu H., Xiong Q., Yi H., Yang H., Mei Z., Zhao Q., Yin Z.-W., Huang M. (2024). Sequential
Co-Reduction of Nitrate and Carbon Dioxide
Enables Selective Urea Electrosynthesis. Nat.
Commun..

[21] Zhang Y., Li Z., Chen K., Yang X., Zhang H., Liu X., Chu K. (2024). Promoting
Electroreduction of CO_2_ and NO_3_
^–^ to Urea via Tandem Catalysis of Zn Single Atoms and
In_2_O_3‑x_. Adv. Energy
Mater..

[22] Li H., Shi J., Zhao K., Zhang L. (2014). Sustainable Molecular
Oxygen Activation
with Oxygen Vacancies on the {001} Facets of BiOCl Nanosheets under
Solar Light. Nanoscale.

[23] Kapilashrami M., Zhang Y., Liu Y.-S., Hagfeldt A., Guo J. (2014). Probing the
Optical Property and Electronic Structure of TiO_2_ Nanomaterials
for Renewable Energy Applications. Chem. rev..

[24] Cao C., Ma D. D., Gu J. F., Xie X., Zeng G., Li X., Han S. G., Zhu Q. L., Wu X. T., Xu Q. (2020). Metal–Organic
Layers Leading to Atomically Thin Bismuthene for Efficient Carbon
Dioxide Electroreduction to Liquid Fuel. Angew.
Chem., Int. Ed..

[25] Greeley J., Jaramillo T. F., Bonde J., Chorkendorff I., Nørskov J. K. (2006). Computational
High-Throughput Screening of Electrocatalytic
Materials for Hydrogen Evolution. Nat. Mater..

[26] Hsieh P.-A., Chen P.-J., Lyu L.-M., Chen S.-Y., Tseng M.-C., Chung M.-Y., Chiang W.-H., Chen J.-L., Kuo C.-H. (2021). Enhanced
Production of Formic Acid in Electrochemical CO_2_ reduction
over Pd-Doped BiOCl Nanosheets. ACS Appl. Mater.
Interfaces.

[27] Xiao Y., Liu D., Yang J., Feng J., Gu W., Qiao L., Ip W. F., Pan H. (2025). Controllable Reconstruction of β-Bi_2_O_3_/Bi_2_O_2_CO_3_ Composite
for Highly Efficient and Durable Electrochemical CO_2_ Conversion. Nano Lett..

[28] Lv W., Bei J., Zhang R., Wang W., Kong F., Wang L., Wang W. (2017). Bi_2_O_2_CO_3_ Nanosheets as Electrocatalysts
for Selective Reduction of CO_2_ to Formate at Low Over potential. ACS Omega.

[29] Xiong J., Cheng G., Li G., Qin F., Chen R. (2011). Well-Crystallized
Square-Like 2D BiOCl Nanoplates: Mannitol-Assisted Hydrothermal Synthesis
and Improved Visible-Light-Driven Photocatalytic Performance. RSC Adv..

[30] Jiang N., Spence J. C. H. (2006). Can Near-Edge
Structure of the Bi L_3_ Edge
Determine the Formal Valence States of Bi?. J. Phys. Condens. Matter.

[31] Liu L., Sun Y., Cui X., Qi K., He X., Bao Q., Ma W., Lu J., Fang H., Zhang P. (2019). Bottom-Up
Growth of Homogeneous Moiré Superlattices in Bismuth Oxychloride
Spiral Nanosheets. Nat. Commun..

[32] Abdullah N., Bahruji H., Rogers S. M., Wells P. P., Catlow C. R. A., Bowker M. (2019). Pd Local Structure and Size Correlations
to the Activity
of Pd/TiO_2_ for Photocatalytic Reforming of Methanol. Phys. Chem. Chem. Phys..

[33] Sang K., Zuo J., Zhang X., Wang Q., Chen W., Qian G., Duan X. (2023). Towards a
Molecular Understanding of the Electronic Metal-Support
Interaction (EMSI) in Heterogeneous Catalysis. Green Energy Environ..

[34] Wang J., Cheng D., Gao M., Li Q., Xin Y., Zhang N., Zhang Z., Yu X., Zhao Z., Zhou K. (2021). Modulation of the Superficial Electronic
Structure via Metal–Support
Interaction for H_2_ Evolution over Pd Catalysts. Chem. Sci..

[35] Jentys A. (1999). Estimation
of Mean Size and Shape of Small Metal Particles by EXAFS. Phys. Chem. Chem. Phys..

[36] Spanjers C.
S., Senftle T. P., van Duin A. C. T., Janik M. J., Frenkel A. I., Rioux R. M. (2014). Illuminating
Surface Atoms in Nanoclusters by Differential
X-Ray Absorption Spectroscopy. Phys. Chem. Chem.
Phys..

[37] Fröhlich N. L., Eggebeen J. J. J., Koper M. T. M. (2024). Measurement
of the Double-Layer Capacitance
of Pt(111) in Acidic Conditions Near the Potential of Zero Charge. Electrochim. Acta.

[38] Fu H. Q., Liu J., Bedford N. M., Wang Y., Wright J., Liu P. F., Wen C. F., Wang L., Yin H., Qi D. (2022). Operando
Converting BiOCl into Bi_2_O_2_(CO_3_)_x_Cl_y_ for Efficient Electrocatalytic
Reduction of Carbon Dioxide to Formate. Nano-Micro
Lett..

[39] Hua Y., Kang D., Huang J., Ni B., Cai W.-B., Jiang K. (2026). Electrified CO_2_-to-HCOOH
Valorization: A Comparative Technical
Analysis on Acidic Flow Cell and Solid-State Electrolyte Cell Reactors. ACS Energy Lett..

