# Supplementary material for: Pd Intercalation in BiOCl Nanosheets Promotes Ambient Electrosynthesis of Urea: Operando Study by Synchrotron X‑ray Spectroscopies
Source: ACS Appl Mater Interfaces. 2026 May 6;18(19):28310–21. doi: 10.1021/acsami.6c03918 (PMC13195566; doi:10.1021/acsami.6c03918)
Supplement: Supplementary file 1 [file am6c03918_si_001.pdf]

Supporting Information

Pd Intercalation in BiOCl Nanosheets Promotes Ambient Electrosynthesis of Urea: Operando Study by Synchrotron X-Ray Spectroscopies

Yu-Chung Chang,[+, §] Shao-Hua Yu,[+, §] Ruei-Hung Juang,[+] Hsing-Ye Chen,[+] Aline Wong Taladua,[¶] Cheng-Shiuan Li,[△] Rey Yonson Capangpangan,[#] Chun-Hong Kuo[*, +, ○, ‡]

[+]*Department of Applied Chemistry, National Yang Ming Chiao Tung University, Hsinchu 300093, Taiwan*

[¶]*Department of Chemistry, Mindanao State University - Iligan Institute of Technology, Iligan 9200, Philippines*

[△]*Green Energy and Environment Research Laboratories, Industrial Technology Research Institute, Hsinchu 310401, Taiwan*

[#]*Department of Physical Sciences and Mathematics, College of Fisheries and Marine Sciences, Mindanao State University at Naawan, Naawan 9023, Philippines*

[○]*Center for Emergent Functional Matter Science, National Yang Ming Chiao Tung University, Hsinchu 300093, Taiwan*

[‡]*National Synchrotron Radiation Research Center, Hsinchu 300092, Taiwan*

Contact Information of the Corresponding Author

*(C.-H. K.) Email: chunhong@nycu.edu.tw

Number of Pages: 18, Number of Tables: 8, Number of Figures: 15

Figure S1. (a) Schematic diagram of an experimental setup for the electrosynthesis of urea. (b) The enlarged view of the marked area in (a), illustrating the detailed structure of a H-cell connected with a standard three-electrode system. The abbreviations of WE, RE, CE, and PEM indicates working electrode, reference electrode, counter electrode, and proton-exchange membrane. ··················S4

Figure S2. (a) The chemical equations for Griess test. (b) UV-Vis absorption spectra of (Z)-4-((4-((2-aminoethyl)amino)naphthalen-1-yl)diazenyl)benzenesulfonamide with a series of known [NO_2^-]. (c) The plot of absorbance in b vs [NO_2^-], which serves as the calibration line for quantitatively estimating [NO_2^-] produced in electrosynthesis of urea. ···S5

Figure S3. (a) The chemcical equations for indophenol blue test. (b) UV-Vis absorption spectra of disodiummono(5-((3-carboxylato-4-oxocyclohexa-2,5-dien-1-ylidene)amino)-2-oxidobenzoate) with a series of known [NH_4^+]. (c) The plot of absorbance in b vs [NO_2^-], which serves as the calibration line for qantitatively estimating [NH_4^+] produced in electrosynthesis of urea. ············S6

Figure S4. The chemcical equations for urease hydrolysis test. The ammonia from urea decompositon is subsequently determined with indophenol blue test. ··S6

Figure S5. Size-distribution histograms of (a) BiOCl and (b) Pd-BiOCl nanosheets. ·················S7

Figure S6. (a, d) AFM images of lying BiOCl and Pd-BiOCl nanosheets. (b, e) The cross-section height profiles along the white lines in (a) and (b). (c, f) SEM images of standing (c) BiOCl and (f) Pd-BiOCl nanosheets. ··S7

Table S1. Measured atomic compositions by ICP-MS. ··S8

Figure S7. k^3-weighted FT-EXAFS spectra and fiiting results of (a) Bi foil, (b) Bi_2O_3, (c) BiOCl, and (d) Pd-BiOCl in Bi L_3-edge, and (e) Pd, and (f) Pd-BiOCl in Pd K-edge. The hollow points represent raw data, and the red lines are the fitting results. ···S8

Figure S8. The selected 2θ range of the SPXRD pattern demonstrates the intercalation of Pd into the BiOCl nanosheet framework. ··S9

Figure S9. (a) Pd K-edge XANES and (b) k^3-weighted FT-EXAFS (phase-uncorrected) of Pd-BiOCl (gray solid), Pd (purple dashed), PdO (green dashed). ··S9

Figure S10. High-resolution X-ray photoelectron spectra of BiOCl and Pd-BiOCl nanosheets. (a) Bi(4f), ΔE_{SO} = 5.3 eV, $4f_{7/2}$: $4f_{5/2}$ area ratio = 4 : 3, (b) O(1s), (c) Cl(2p), ΔE_{SO} = 5.3 eV, $2p_{3/2}$: $2p_{1/2}$ area ratio = 2 : 1, (d) Pd(3d), ΔE_{SO} = 5.26 eV, $3d_{5/2}$: $3d_{3/2}$ area ratio = 3 : 2. Spectra were referenced to Au $4f_{7/2}$ = 84 eV. ···S10

Table S2. The best-fit EXAFS parameters of BiOCl and Pd-BiOCl nanosheets and their relative references. ···S11

Table S3. EIS fitting results of BiOCl-WE, Pd-BiOCl-WE, and Pd/C-WE. ·························S11

Figure S11. CVs obtained by scanning in the ranges of OCP ± 0.05 V (vs RHE) at different scan rates (r) of 20, 40, 60, 80, and 100 mV/s for (a) BiOCl-WE, (b) Pd-BiOCl-WE, and (c) Pd/C-WE. ·······S12

Table S4. Collected C_{dl} and ECSA of BiOCl-WE, Pd-BiOCl-WE, and Pd/C-WE. ···················S13

Table S5. Total F.E. (%) of BiOCl-WE, Pd-BiOCl-WE, and Pd/C-WE in urea electrosynthesis.····S13

Table S6. Yield rates normalized by the catalyst weights (Y_{catal}) of BiOCl-WE, Pd-BiOCl-WE, and Pd/C-WE for urea electrosynthesis. ···S14

Figure S12. Faradaic efficiencies (F.E.) of products formed from (a-c) CO_2RR and (d-f) NO_3RR over the (a, d) BiOCl-WE, (b, e) Pd-BiOCl-WE, and (c, f) Pd/C-WE. ······S14
Table S7. Total F.E. (%) of BiOCl-WE, Pd/BiOCl-WE, and Pd/C-WE in CO_2RR. ······S15
Table S8. Total F.E. (%) of BiOCl, Pd-BiOCl, and Pd/C in NO_3RR. ······S15
Figure S13. Durability test for urea electrosynthesis over Pd-BiOCl and BiOCl nanosheets at −0.3 V for 100 hours. ······S16
Figure S14. Side-view schemes of a $Bi_2O_2CO_3$ unit cell along <001> and <111> directions. ······S17
Figure S15. The selected 2θ range of SPXRD where reveals the generation of Bi^0 over the BiOCl nanosheets under negative potentials. ······S18

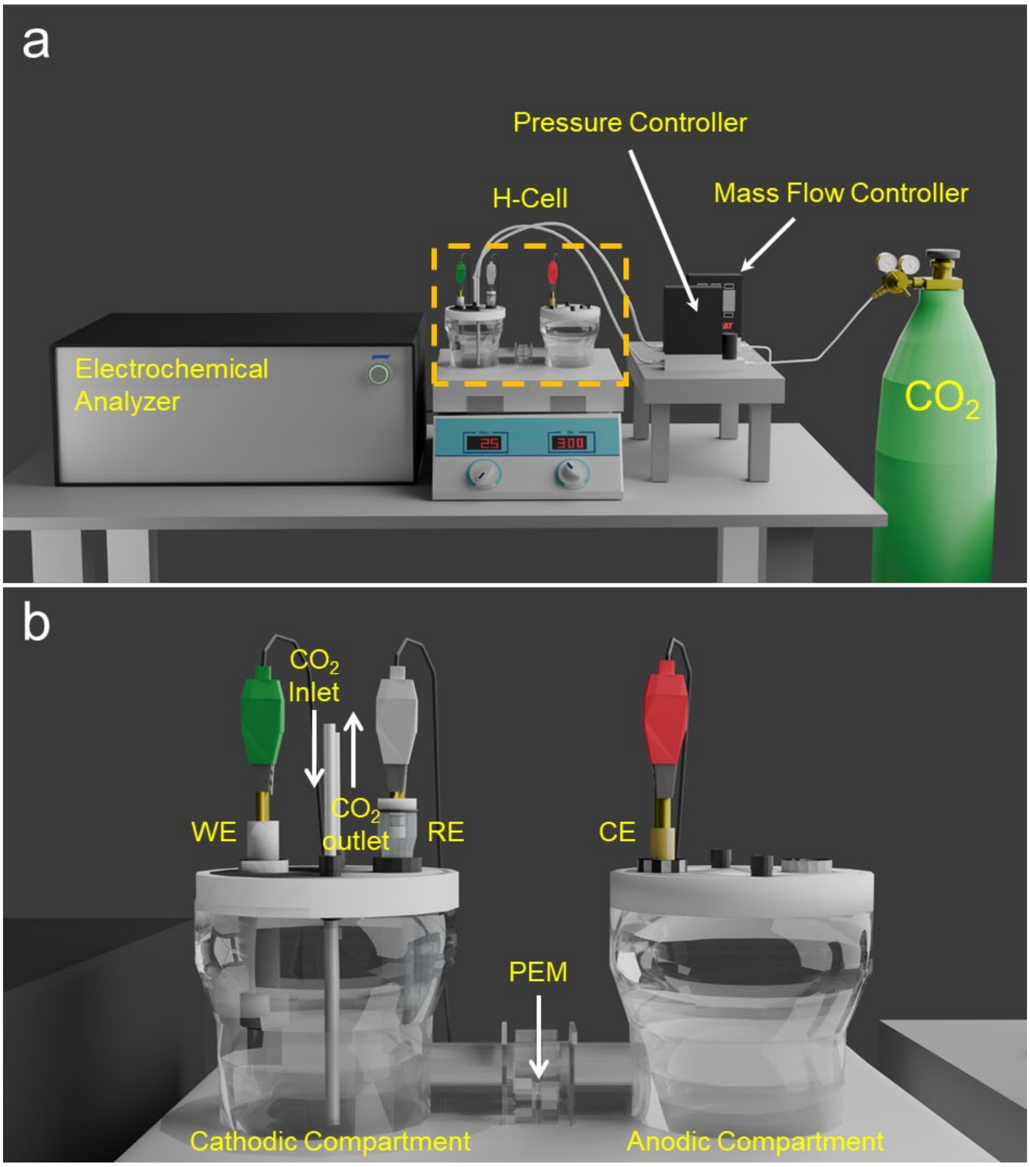

Figure S1. (a) Schematic diagram of an experimental setup for the electrosynthesis of urea. (b) The enlarged view of the marked area in (a), illustrating the detailed structure of a H-cell connected with a standard three-electrode system. The abbreviations of WE, RE, CE, and PEM indicates working electrode, reference electrode, counter electrode, and proton-exchange membrane.

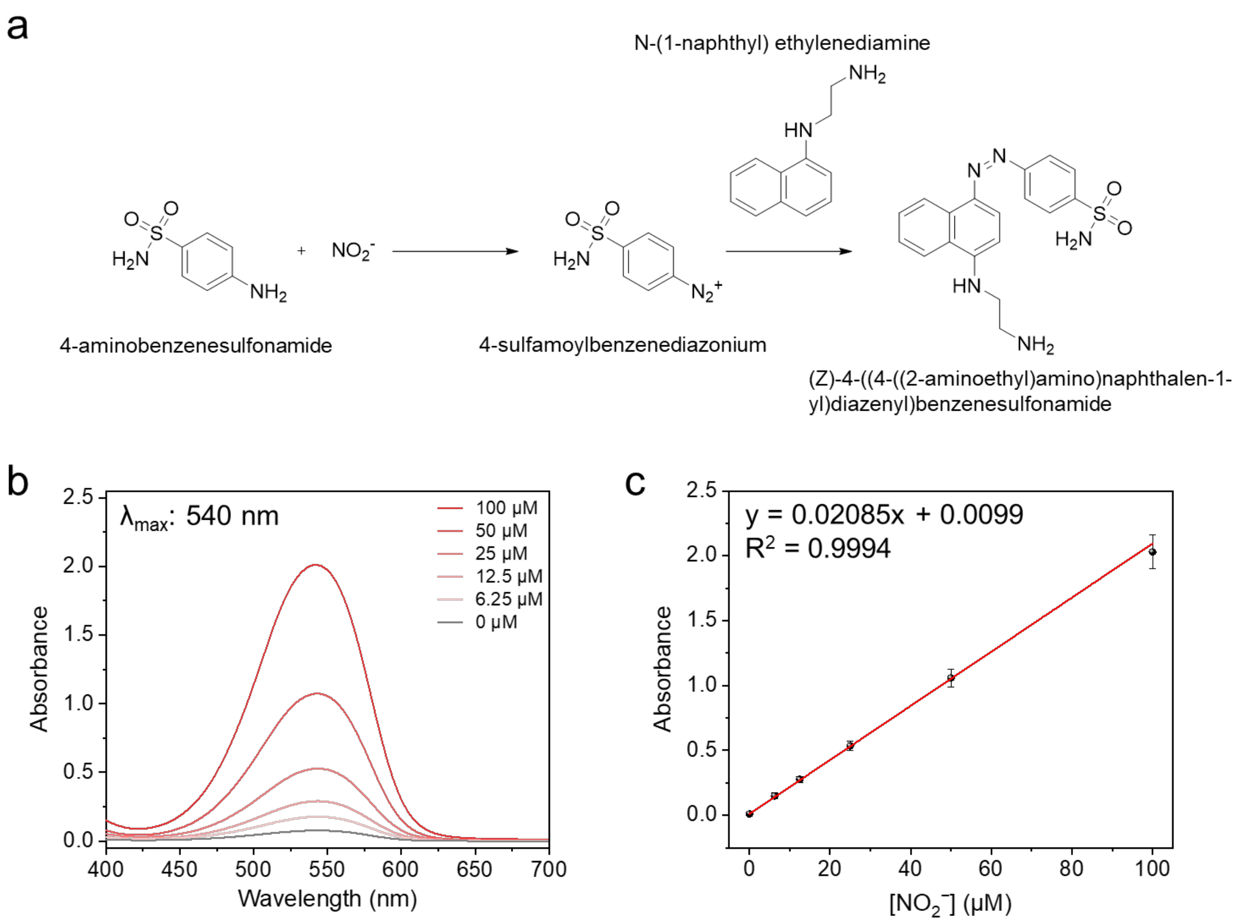

Figure S2. (a) The chemical equations for Griess test. (b) UV-Vis absorption spectra of (Z)-4-((4-((2-aminoethyl)amino)naphthalen-1-yl)diazenyl)benzenesulfonamide with a series of known [NO_2^-]. (c) The plot of absorbance in b vs [NO_2^-], which serves as the calibration line for quantitatively estimating [NO_2^-] produced in electrosynthesis of urea.

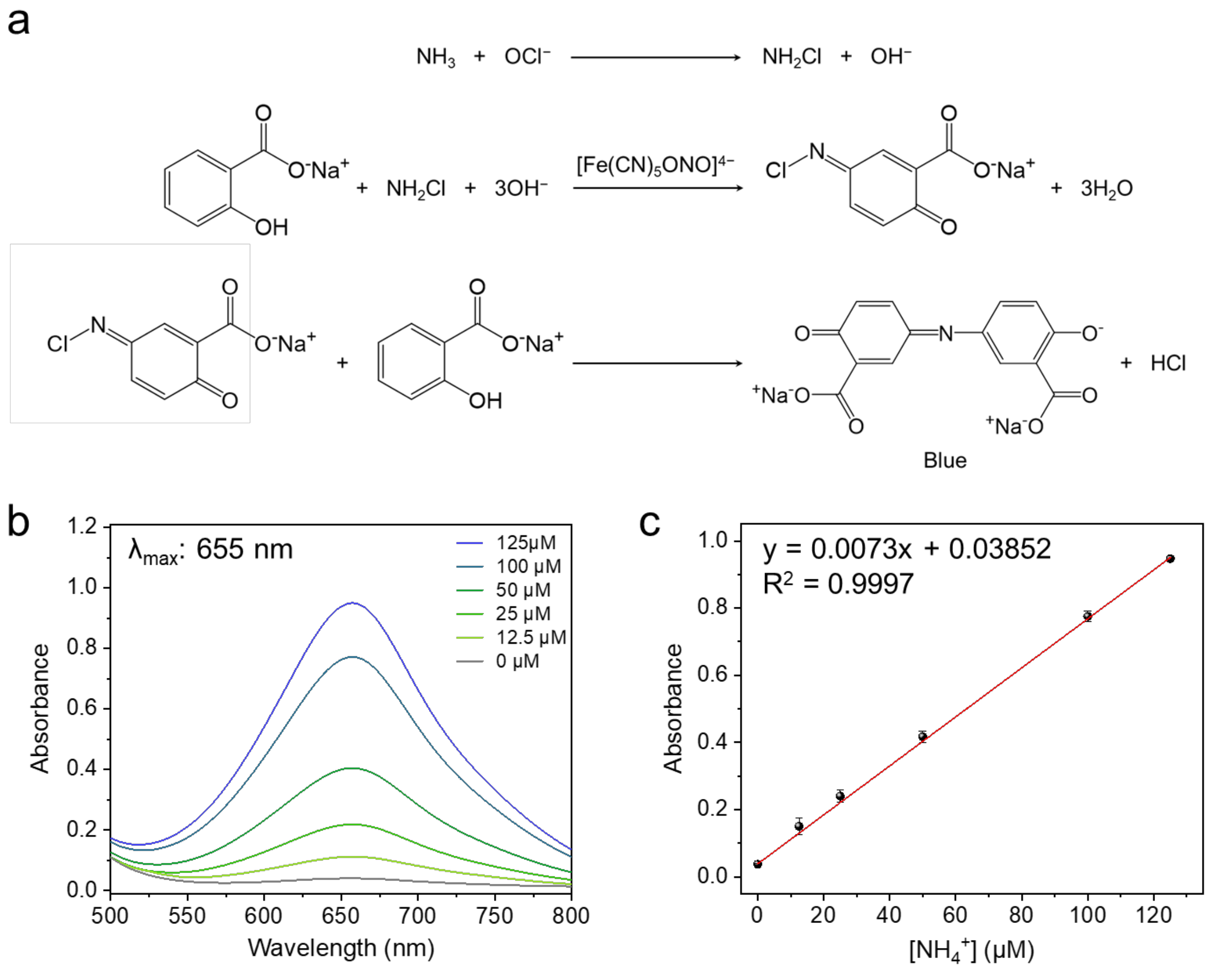

Figure S3. (a) The chemcical equations for indophenol blue test. (b) UV-Vis absorption spectra of disodiummono(5-((3-carboxylato-4-oxocyclohexa-2,5-dien-1-ylidene)amino)-2-oxidobenzoate) with a series of known [NH_4^+]. (c) The plot of absorbance in b vs [NO_2^-], which serves as the calibration line for qantitatively estimating [NH_4^+] produced in electrosynthesis of urea.

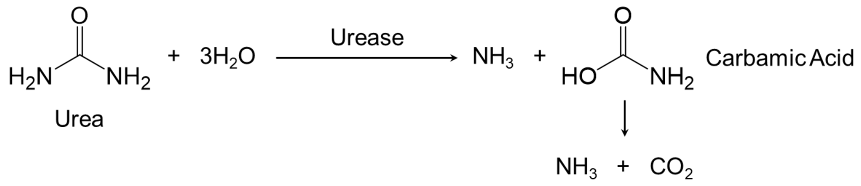

Figure S4. The chemcical equations for urease hydrolysis test. The ammonia from urea decompositon is subsequently determined with indophenol blue test.

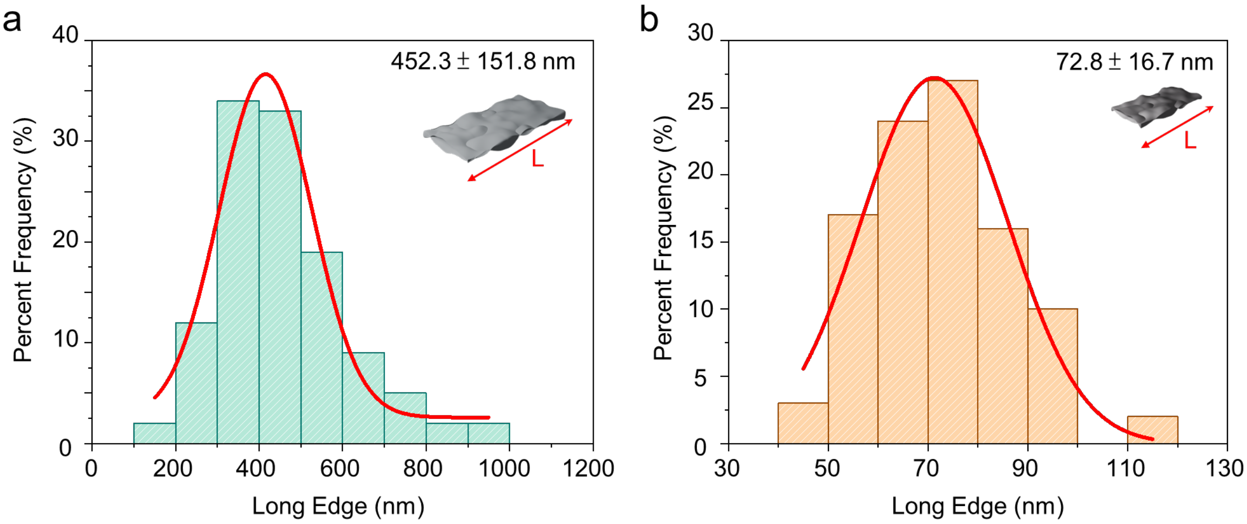

Figure S5. Size-distribution histograms of (a) BiOCl and (b) Pd-BiOCl nanosheets.

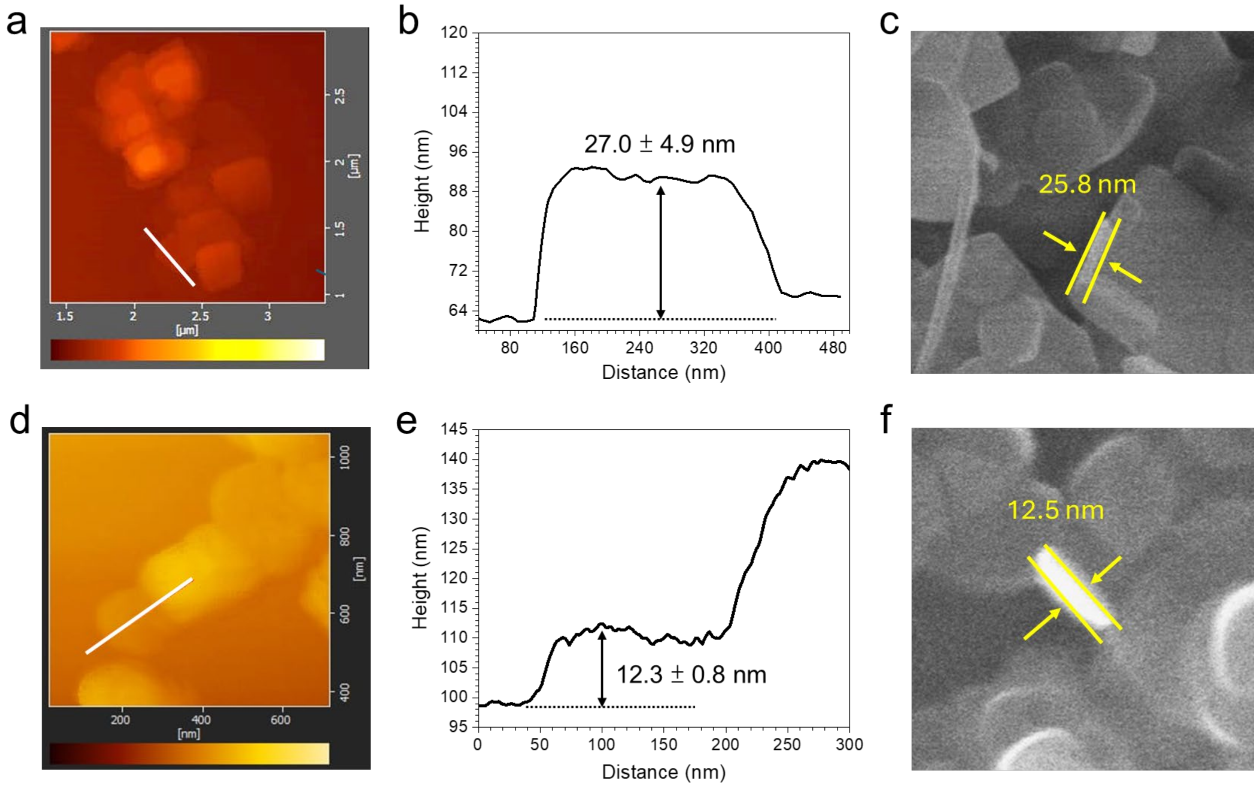

Figure S6. (a, d) AFM images of lying BiOCl and Pd-BiOCl nanosheets. (b, e) The cross-section height profiles along the white lines in (a) and (b). (c, f) SEM images of standing (c) BiOCl and (f) Pd-BiOCl nanosheets.

Table S1. Measured atomic compositions by ICP-MS

Sample	Bi (ppm)	Pd (ppm)	Pd (%)
Pd-BiOCl	641183 ± 3643	12092 ± 88	1.89
BiOCl	696274 ± 3707	N/A	0

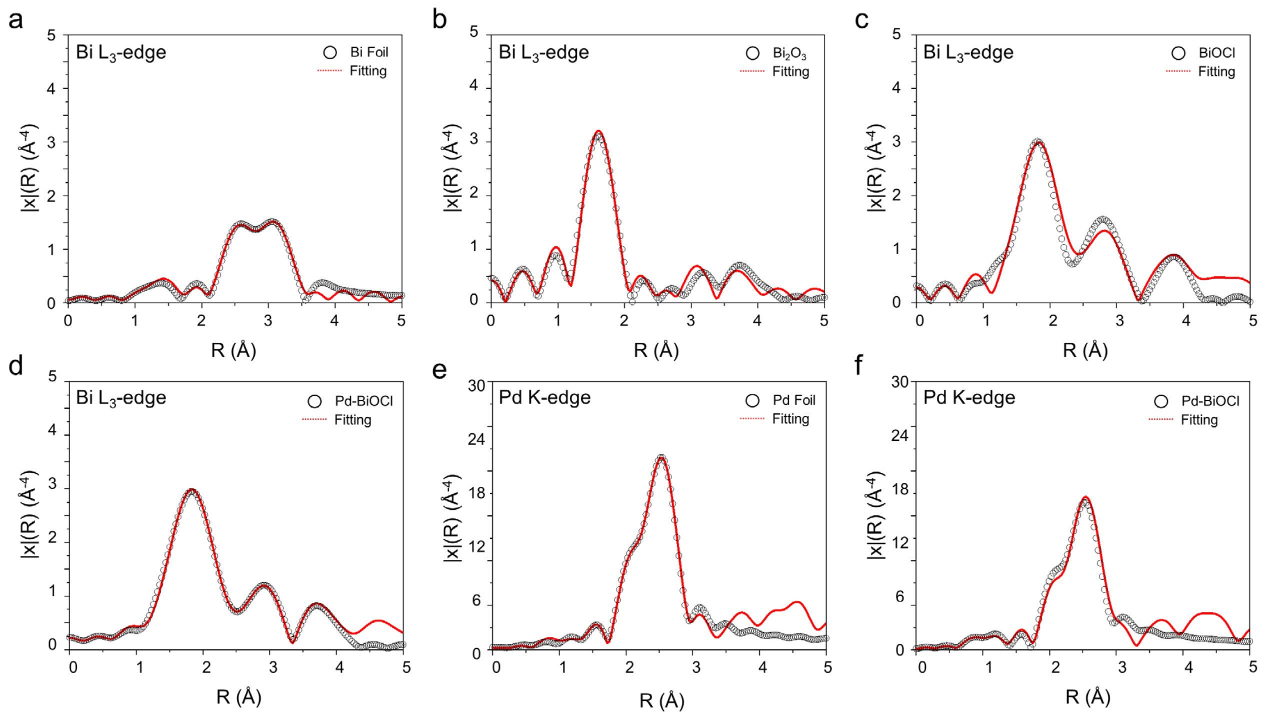

Figure S7. k^3-weighted FT-EXAFS spectra and fiiting results of (a) Bi foil, (b) Bi_2O_3, (c) BiOCl, and (d) Pd-BiOCl in Bi L_3-edge, and (e) Pd, and (f) Pd-BiOCl in Pd K-edge. The hollow points represent raw data, and the red lines are the fitting results.

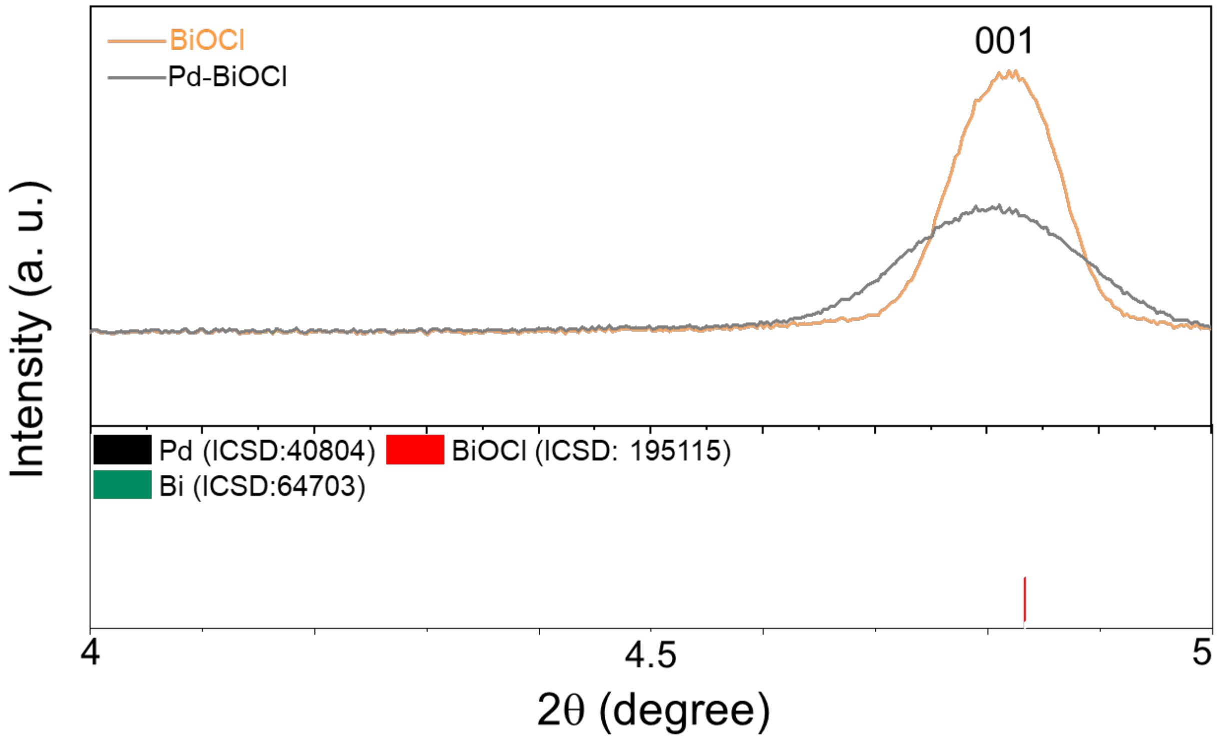

Figure S8. The selected 2θ range of the SPXRD pattern demonstrates the intercalation of Pd into the BiOCl nanosheet framework.

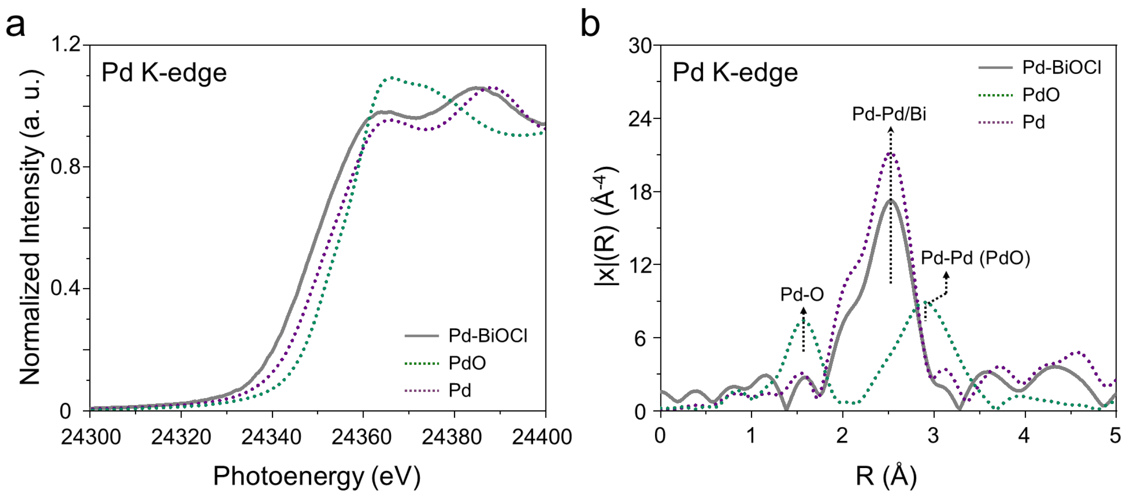

Figure S9. (a) Pd K-edge XANES and (b) k^3-weighted FT-EXAFS (phase-uncorrected) of Pd-BiOCl (gray solid), Pd (purple dashed), PdO (green dashed).

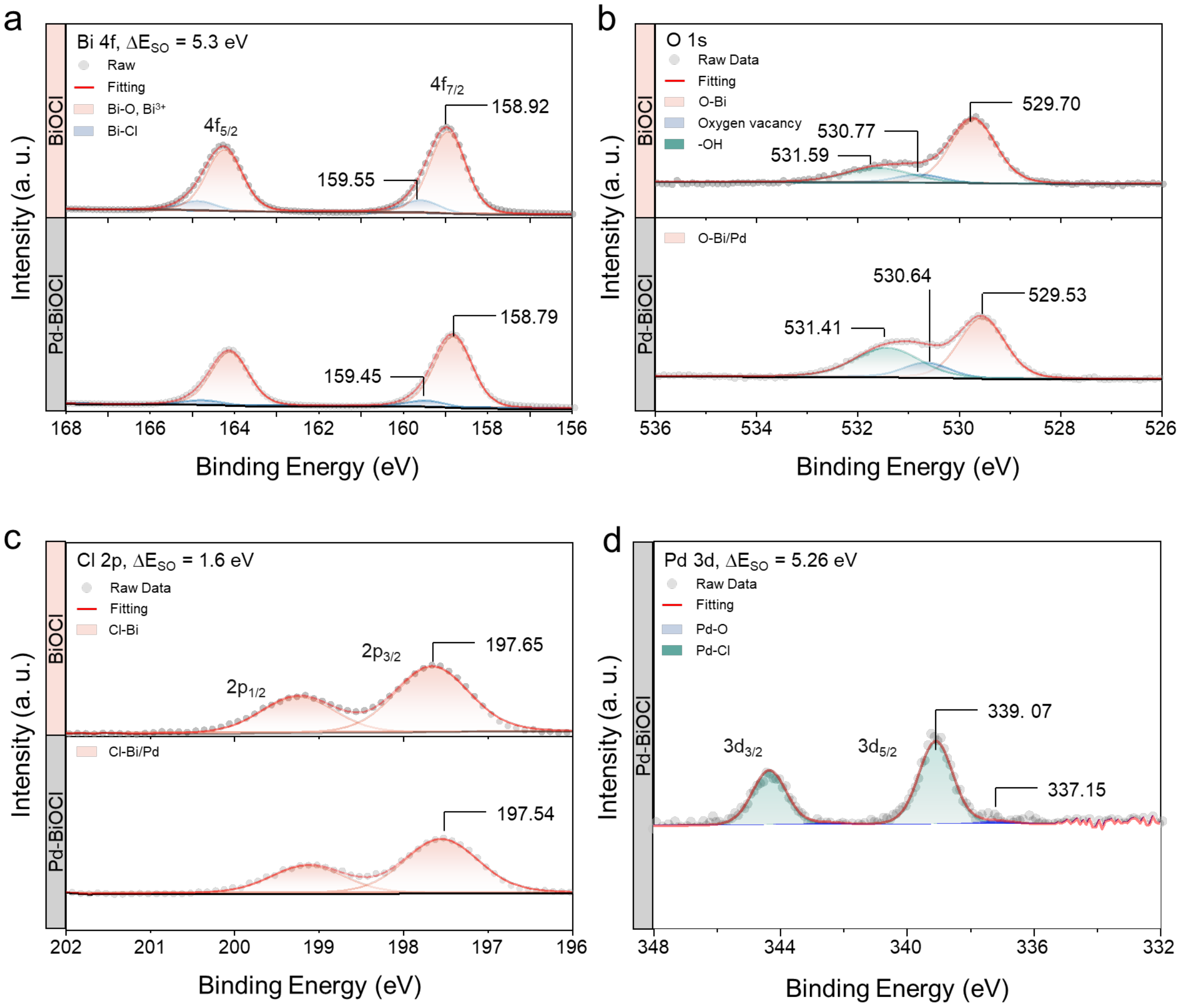

Figure S10. High-resolution X-ray photoelectron spectra of BiOCl and Pd-BiOCl nanosheets. (a) Bi(4f), ΔE_{SO} = 5.3 eV, $4f_{7/2}$: $4f_{5/2}$ area ratio = 4 : 3, (b) O(1s), (c) Cl(2p), ΔE_{SO} = 5.3 eV, $2p_{3/2}$: $2p_{1/2}$ area ratio = 2 : 1, (d) Pd(3d), ΔE_{SO} = 5.26 eV, $3d_{5/2}$: $3d_{3/2}$ area ratio = 3 : 2. Spectra were referenced to Au $4f_{7/2}$ = 84 eV.

Table S2. The best-fit EXAFS parameters of BiOCl and Pd-BiOCl nanosheets and their relative references.

	Scattering Path	N	σ^2(Å^2)	R(Å)	R_f
Bi Foil	Bi-Bi	3	0.004	3.06	0.01
	Bi-Bi	3	0.014	3.58	
Pd Foil	Pd-Pd	12	0.004	2.74	0.002
Bi_2O_3	Bi-O	3.42	0.005	2.11	0.03
	Bi-Bi	3.33	0.008	3.81	
BiOCl (as-prepared sample)	Bi-O	4.02	0.007	2.27	0.01
	Bi-Cl	4.11	0.015	3.04	
	Bi-Bi	6.5	0.009	3.74	
Pd-BiOCl (as-prepared sample)	Bi-O	3.65	0.009	2.28	0.013
	Bi-Cl	3.77	0.017	3.05	
	Bi-Bi (Bi-O-Bi)	6.03	0.007	3.75	
	Bi-Pd	1.73	0.005	2.58	
	Pd-O	2.73	0.006	2.58	0.02
	Pd-Bi	1.1	0.003	2.58	
	Pd-Pd	6.4	0.003	2.73	

Fitting was done across the *k* range of 2.7 to 10.3 Å^{-1} and the *R* range of 1 to 4.25 Å for all samples. Where N is the coordination number, R is the distance between the absorber and backscatter atoms, σ^2 is the Debye-Waller factor, and R_f is the R-factor characterizing the goodness of fitting. S_o^2 was fixed to 0.73 and 0.638 as determined from Bi (ICSD: 64703) and Pd (ICSD:40804) metal foil fitting. Error bounds (accuracies) characterizing the structural parameters obtained by EXAFS data analysis are estimated to be as follows: N, ±20%; R, ±1%; and σ^2, ±20%.

Table S3. EIS fitting results of BiOCl-WE, Pd-BiOCl-WE, and Pd/C-WE.

Sample	R_s (Ω)	R_{ct} (Ω)	CPE_{T1} (mFs^{a-1})	CPE_{P1} (= a)
BiOCl-WE	30.42	225.9	0.00093	0.89125
Pd-BiOCl-WE	31.38	136.9	0.00016	0.86527
Pd/C-WE	29.17	79.97	0.00130	0.79532

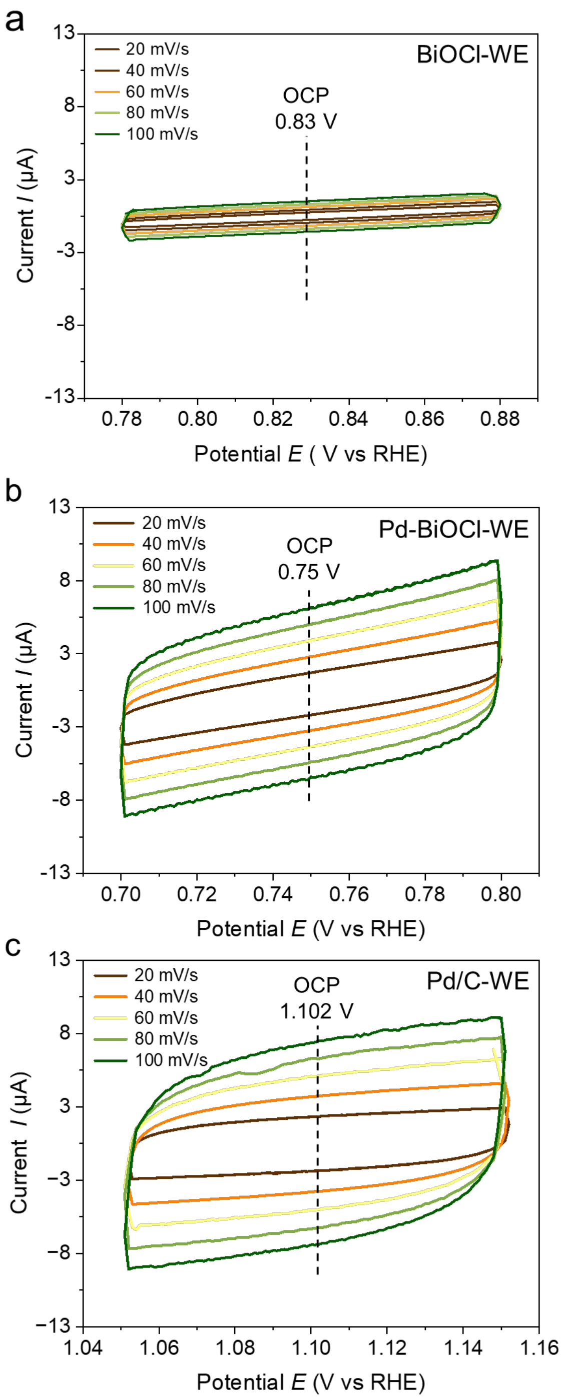

Figure S11. CVs obtained by scanning in the ranges of OCP ± 0.05 V (vs RHE) at different scan rates (r) of 20, 40, 60, 80, and 100 mV/s for (a) BiOCl-WE, (b) Pd-BiOCl-WE, and (c) Pd/C-WE.

Table S4. Collected C_{dl} and ECSA of BiOCl-WE, Pd-BiOCl-WE, and Pd/C-WE.

**Sample**	**C_{dl} (μF)**	**ECSA (cm²)**
BiOCl-WE	0.010	0.250
Pd–BiOCl-WE	0.054	1.357
Pd/C-WE	0.062	1.570

Table S5. Total F.E. (%) of BiOCl-WE, Pd-BiOCl-WE, and Pd/C-WE in urea electrosynthesis.

Sample	Potential (V vs RHE)	NO_2^-	NH_4^+	Urea	$HCOO^-$	CO	H_2
Pd-BiOCl-WE	-0.2	43.97 ± 3.31	28.09 ± 2.53	7.23 ± 0.64	2.23 ± 1.25	0	8.29 ± 1.62
	-0.3	46.20 ± 1.14	32.02 ± 2.94	12.43 ± 1.02	4.99 ± 2.4	0	9.28 ± 1.23
	-0.4	32.34 ± 1.29	33.93 ± 3.32	5.64 ± 0.77	5.18 ± 1.96	0	10.94 ± 2.23
	-0.5	25.77 ± 1.70	38.59 ± 2.16	4.01 ± 1.12	4.44 ± 1.19	0	14.36 ± 1.71
	-0.6	23.31 ± 2.82	42.67 ± 1.57	3.4 ± 0.51	3.57 ± 0.81	0	16.54 ± 1.84
BiOCl-WE	-0.2	40.22 ± 1.43	18.25 ± 0.32	1.44 ± 0.43	0	0	10.23 ± 1.92
	-0.3	39.79 ± 0.89	18.52 ± 0.95	6.99 ± 1.62	1.22 ± 0.62	0	12.65 ± 0.79
	-0.4	28.85 ± 1.22	21.62 ± 1.39	4.54 ± 0.86	1.91 ± 0.94	0	15.61 ± 1.06
	-0.5	24.07 ± 3.79	28.94 ± 1.12	2.72 ± 1.56	2.38 ± 1.18	0	18.45 ± 0.98
	-0.6	16.97 ± 0.67	35.62 ± 0.75	1.21 ± 0 .40	3.57 ± 1.61	0	18.19 ± 1.67
Pd/C-WE	-0.2	31.74 ± 0.57	9.63 ± 0.65	0	0.83 ± 0.31	0	21.69 ± 1.09
	-0.3	32.68 ±2 .94	10.10 ± 1.09	0	3.91 ± 0.98	0	20.67 ± 1.18
	-0.4	33.07 ± 1.15	12.27 ± 0.55	0	6.1 ± 1.32	0	24.22 ± 1.63
	-0.5	29.04 ± 1.60	13.25 ± 0.94	0	8.85 ± 1.83	0	30.54 ± 0.85
	-0.6	22.91 ± 1.41	22.81 ± 1.56	0	1.3 ± 0.47	8.05 ± 1.28	36.33 ± 1.06

The electrolyte consists of 0.1 M $KHCO_3$ and 0.1 M KNO_3 with 20 SCCM flow of CO_2 purged throughout the reaction.

Table S6. Yield rates normalized by the catalyst weights (Y_{catal}) of BiOCl-WE, Pd-BiOCl-WE, and Pd/C-WE for urea electrosynthesis.

Sample	Potential (V vs RHE)	Urea Y_{catal} ($\mu mol \cdot h^{-1} \cdot mg^{-1}$)
Pd-BiOCl-WE	-0.2	0.0339 ± 0.0036
	-0.3	0.1155 ± 0.0135
	-0.4	0.0561 ± 0.0083
	-0.5	0.0613 ± 0.0180
	-0.6	0.0748 ± 0.0133
BiOCl-WE	-0.2	0.0046 ± 0.0012
	-0.3	0.0403 ± 0.0103
	-0.4	0.0312 ± 0.0055
	-0.5	0.0286 ± 0.0156
	-0.6	0.0176 ± 0.0061

The electrolyte consists of 0.1 M $KHCO_3$ and 0.1 M KNO_3 with 20 SCCM flow of CO_2 purged throughout the reaction.

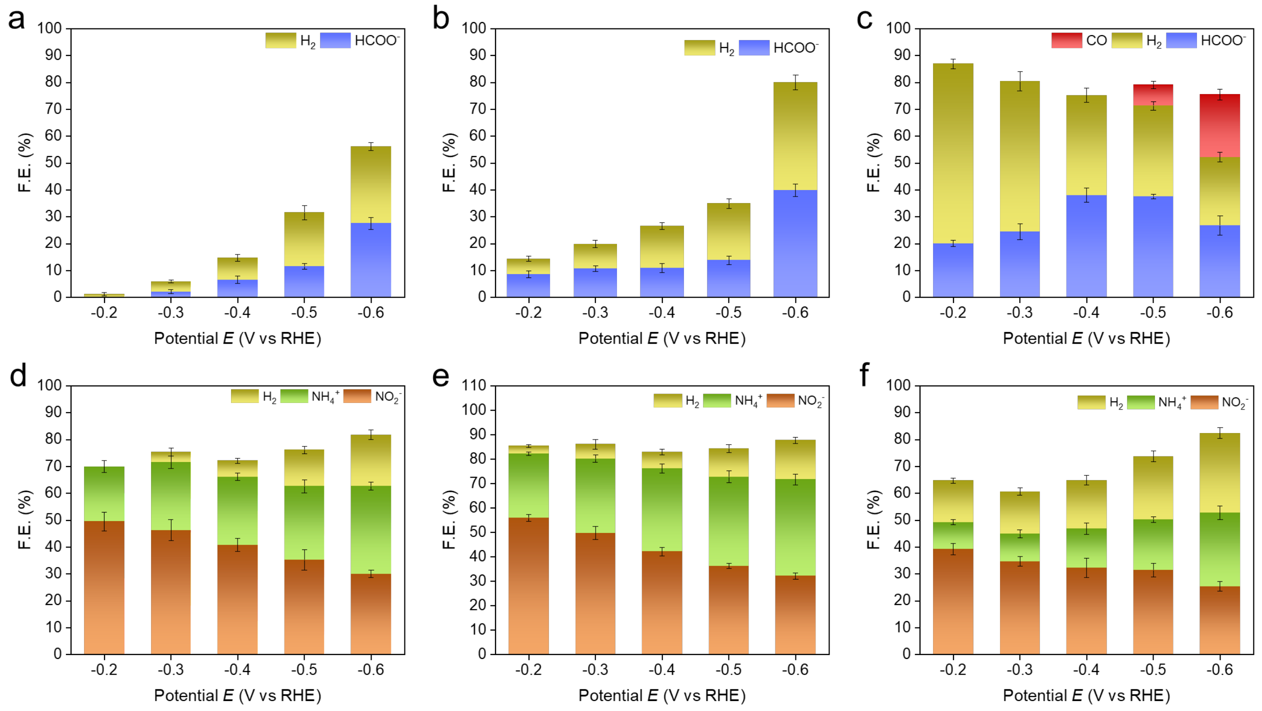

Figure S12. Faradaic efficiencies (F.E.) of products formed from (a-c) CO_2RR and (d-f) NO_3RR over the (a, d) BiOCl-WE, (b, e) Pd-BiOCl-WE, and (c, f) Pd/C-WE.

Table S7. Total F.E. (%) of BiOCl-WE, Pd/BiOCl-WE, and Pd/C-WE in CO_2RR.

Sample	Potential (V vs RHE)	$HCOO^-$	CO	H_2
Pd/BiOCl	-0.2	8.71 ± 1.24	0	5.69 ± 0.96
	-0.3	10.83 ± 1.06	0	9.11 ± 1.35
	-0.4	10.99 ± 1.74	0	15.61 ± 1.20
	-0.5	13.84 ± 1.52	0	21.13 ± 1.75
	-0.6	39.93 ± 2.27	0	40.08 ± 2.68
BiOCl	-0.2	0	0	1.32 ± 0.60
	-0.3	2.18 ± 0.78	0	3.75 ± 0.51
	-0.4	6.58 ± 1.39	0	8.12 ± 1.29
	-0.5	11.69 ± 1.06	0	19.93 ± 2.66
	-0.6	27.56 ± 2.27	0	28.67 ± 1.45
Pd/C	-0.2	20.14 ± 1.26	0	66.85 ± 1.82
	-0.3	24.55 ± 2.97	0	55.90 ± 3.55
	-0.4	38.08 ± 2.66	0	37.19 ± 2.59
	-0.5	37.51 ± 0.83	7.83 ± 1.37	33.80 ± 1.62
	-0.6	26.82 ± 3.51	23.30 ± 1.98	25.43 ± 1.86

The electrolyte consists of 0.1 M $KHCO_3$ with 20 SCCM flow of Ar purged throughout the reaction.

Table S8. Total F.E. (%) of BiOCl, Pd-BiOCl, and Pd/C in NO_3RR.

Sample	Potential (V vs RHE)	NO_2^-	NH_4^+	H_2
Pd/BiOCl	-0.2	55.99 ± 1.31	26.20 ± 0.71	3.23 ± 0.57
	-0.3	49.78 ± 2.68	30.43 ± 1.45	5.86 ± 1.94
	-0.4	42.21 ± 1.72	33.99 ± 1.94	6.83 ± 1.15
	-0.5	36.35 ± 0.97	36.42 ± 2.40	11.59 ± 1.62
	-0.6	32.21 ± 1.24	39.45 ± 2.24	16.06 ± 1.28
BiOCl	-0.2	49.55 ± 3.54	20.43 ± 2.18	0
	-0.3	46.31 ± 3.98	25.24 ± 2.31	3.78 ± 1.47
	-0.4	40.78 ± 2.42	25.38 ± 1.39	6.05 ± 0.96
	-0.5	35.24 ± 3.79	27.41 ± 2.44	13.54 ± 1.35
	-0.6	30.04 ± 1.33	32.69 ± 1.48	19.09 ± 1.81
Pd/C	-0.2	39.20 ± 2.08	10.08 ± 0.98	15.51 ± 0.96
	-0.3	34.73 ± 1.73	10.21 ± 1.52	15.73 ± 1.31
	-0.4	32.28 ± 3.65	14.65 ± 2.07	18.01 ± 1.83
	-0.5	31.43 ± 2.60	18.82 ± 1.12	23.57 ± 2.04
	-0.6	25.41 ± 1.76	27.34 ± 2.58	29.72 ± 1.93

The electrolyte consists of 0.1 M $KHCO_3$ with 20 SCCM flow of CO_2 purged throughout the reaction.

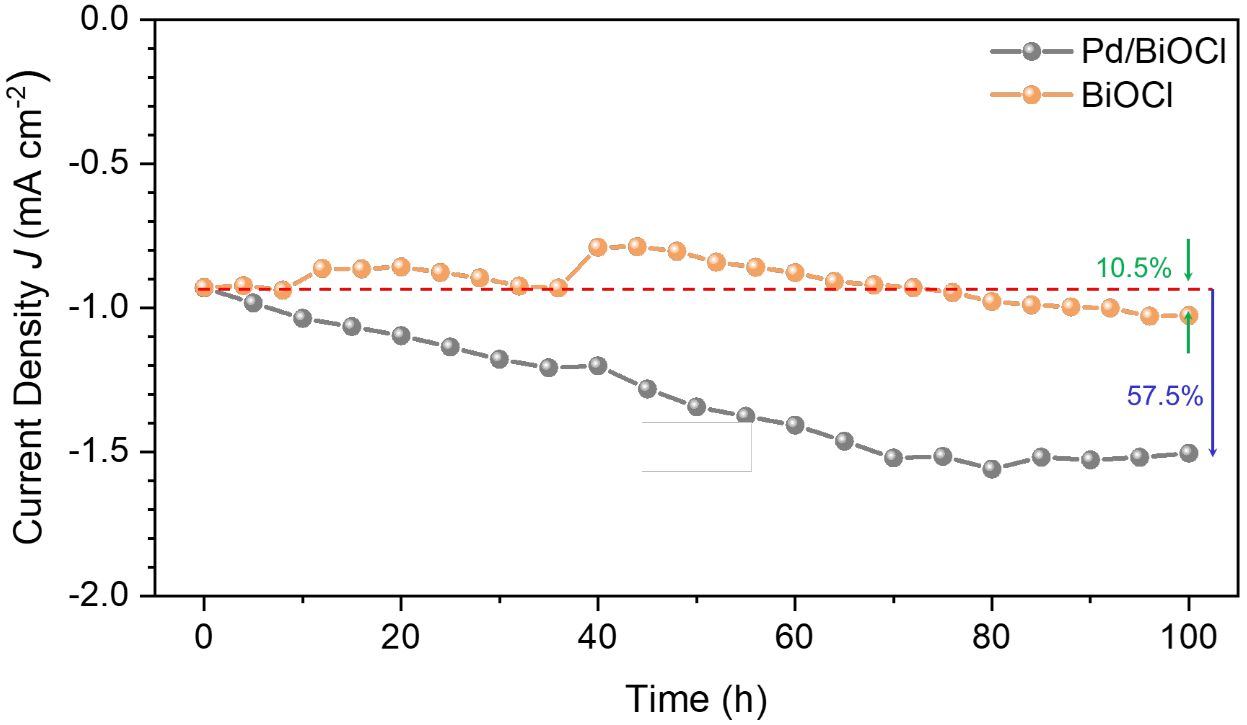

Figure S13. Durability test for urea electrosynthesis over Pd-BiOCl and BiOCl nanosheets at −0.3 V for 100 hours.

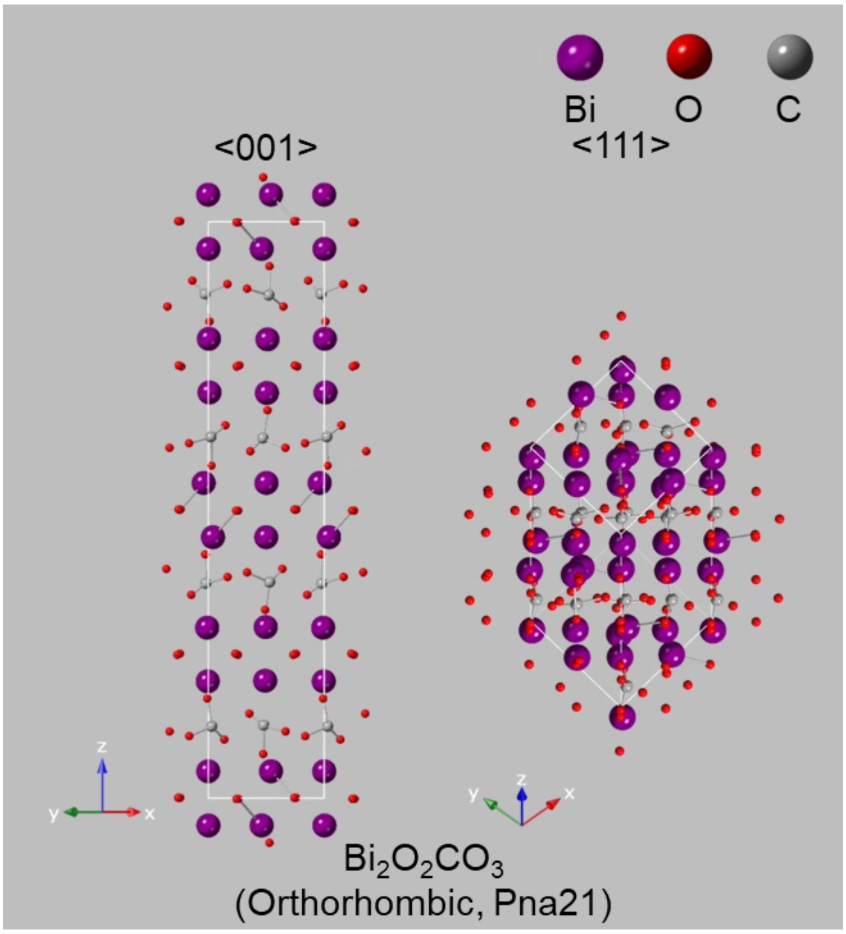

Figure S14. Side-view schemes of a $Bi_2O_2CO_3$ unit cell along <001> and <111> directions.

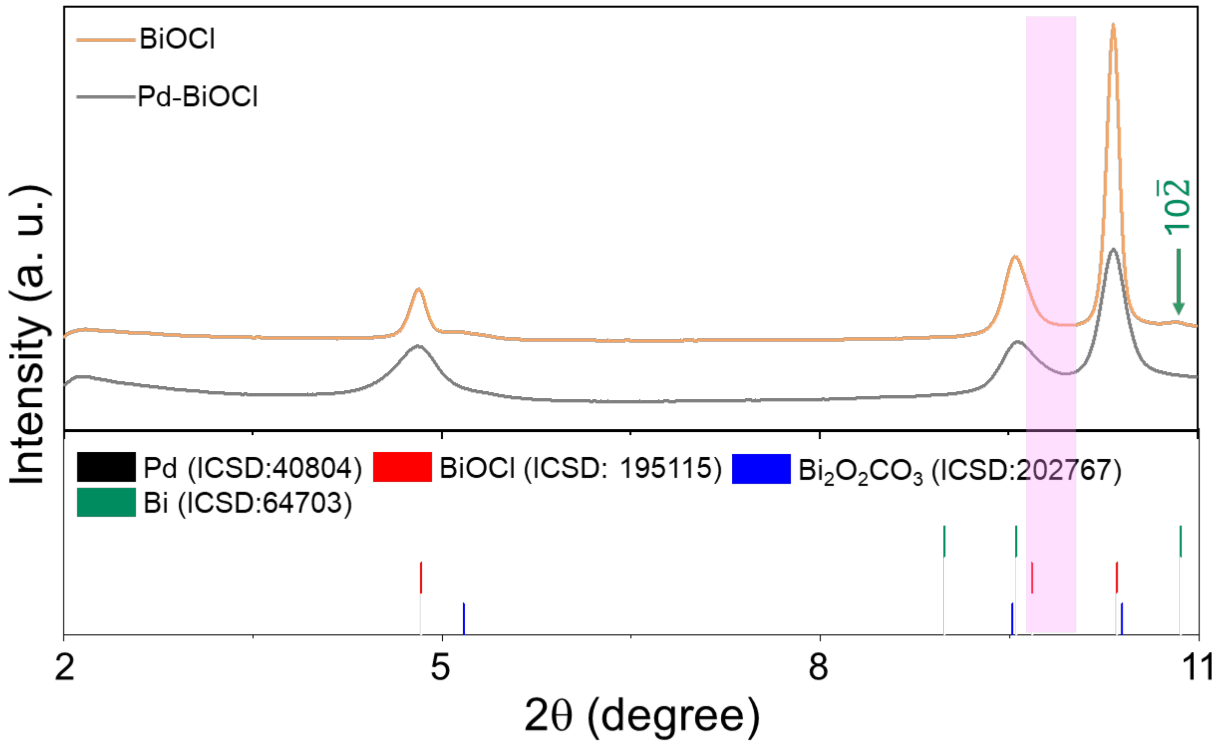

Figure S15. The selected 2θ range of SPXRD where reveals the generation of Bi^0 over the BiOCl nanosheets under negative potentials.
